# Comparison of Computational Strategies for the Calculation of the Electronic Coupling in Intermolecular Energy and Electron Transport Processes

**DOI:** 10.1021/acs.jpca.3c05998

**Published:** 2023-12-12

**Authors:** Xavier López, Aitor Sánchez-Mansilla, Carmen Sousa, Tjerk P. Straatsma, Ria Broer, Coen de Graaf

**Affiliations:** † Departament de Química Física i Inorgànica, Universitat Rovira i Virgili, C. Marcel·lí Domingo 1, 43007 Tarragona, Spain; ‡ Departament de Ciència de Materials i Química Física and Institut de Química Teòrica i Computacional, Universitat de Barcelona, C. Martí i Franquès, 08028 Barcelona, Spain; § National Center for Computational Sciences, Oak Ridge National Laboratory, Oak Ridge, Tennessee 37831-6373, United States; ∥ Department of Chemistry and Biochemistry, University of Alabama, Tuscaloosa, Alabama 35487-0336, United States; ⊥ Zernike Institute of Advanced Materials, University of Groningen, Nijenborgh 4, 9747 AG Groningen, Netherlands; # ICREA, Pg. Lluís Companys 23, 08010 Barcelona, Spain

## Abstract

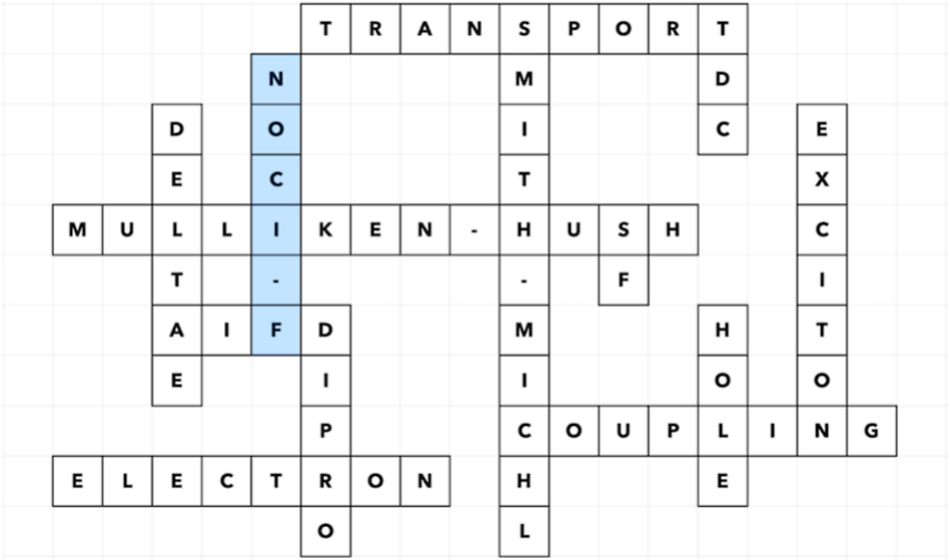

Electronic couplings in intermolecular electron and energy transfer processes calculated by six different existing computational techniques are compared to nonorthogonal configuration interaction for fragments (NOCI-F) results. The paper addresses the calculation of the electronic coupling in diketopyrrolopyrol, tetracene, 5,5′-difluoroindigo, and benzene–Cl for hole and electron transport, as well as the local exciton and singlet fission coupling. NOCI-F provides a rigorous computational scheme to calculate these couplings, but its computational cost is rather elevated. The here-considered ab initio Frenkel–Davydov (AIFD), Dimer projection (DIPRO), transition dipole moment coupling, Michl–Smith, effective Hamiltonian, and Mulliken–Hush approaches are computationally less demanding, and the comparison with the NOCI-F results shows that the NOCI-F results in the couplings for hole and electron transport are rather accurately predicted by the more approximate schemes but that the NOCI-F exciton transfer and singlet fission couplings are more difficult to reproduce.

## 1 Introduction

The coupling between initial and final quantum states plays an important role in the theoretical description of the efficiency of bio- and physicochemical processes such as light harvesting, photoluminescence, charge transport, and exciton dispersion, among many others. For not too strong coupling, the rate of electron and energy transfer in these processes is governed by Fermi’s golden rule
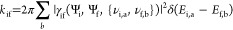
1where γ_if_ is the coupling between initial and final states. It involves electronic degrees of freedom (Ψ_i_ and Ψ_f_) and vibrational degrees of freedom; {ν_i_} and {ν_f_} represent the collection of vibrational states before and after the transfer process. *E*_i,a_ is the energy of the initial state a and *E*_b,f_ of the final state b. A Boltzmann distribution can be used for the population of the initial states ν_i,a_ to take into account temperature effects in an approximate manner. Furthermore, the Condon approximation decouples the vibrational degrees of freedom from the electronic ones and implies that the expression for *k*_if_ can be factorized
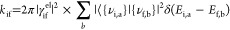
2where the electronic coupling γ_if_^el^ is defined as
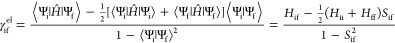
3where Ψ_i_ and Ψ_f_ are normalized wave functions of the initial and final states. The strength of the electronic coupling is difficult to determine experimentally but can be studied in great detail by computational approaches. Such studies provide information not only on the magnitude of the coupling but also on the physical mechanisms that control the coupling and hence can lead to new insights and design rules for new materials. The present study compares different approaches that have been used to calculate γ_if_^el^ to our nonorthogonal configuration interaction for fragments (NOCI-F) in order to address the adequacy of more approximate (and computationally less demanding) methods to obtain useful insights on the couplings in intermolecular electron and energy transfer processes.

**Figure 1:**
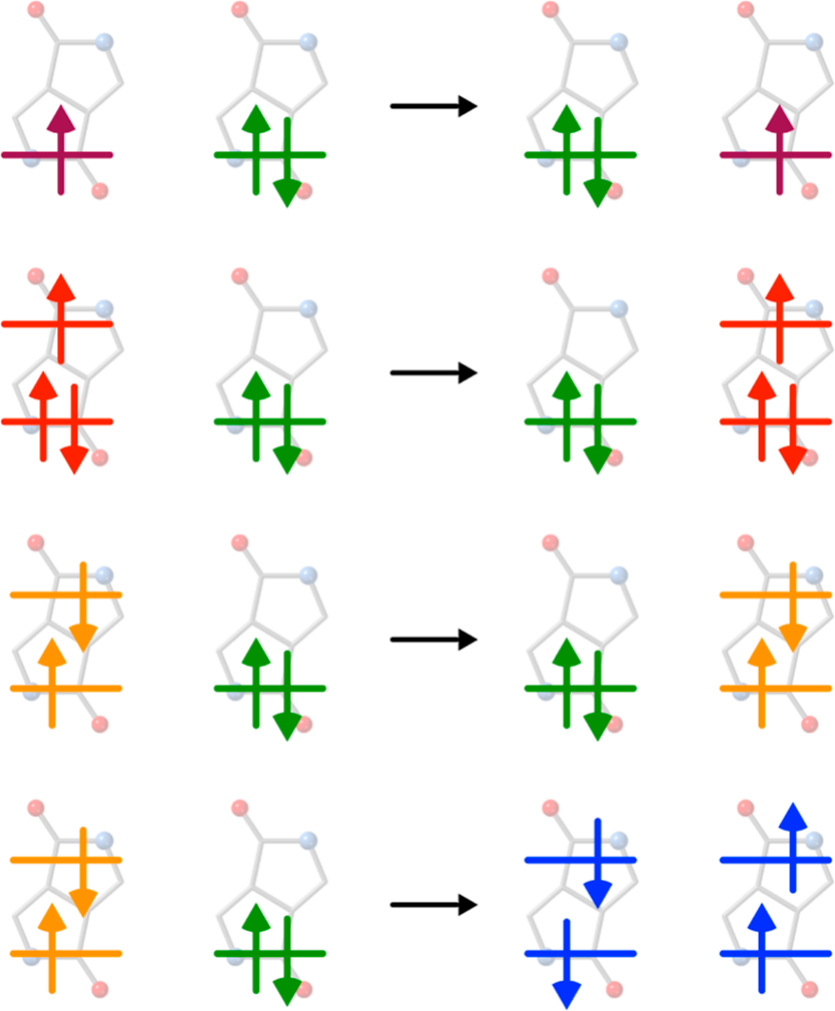
Schematic representation of the initial and final electronic configurations for (from top to bottom) hole transport, electron transport, exciton dispersion, and singlet fission.

We selected four different processes relevant to organic conductors and singlet fission materials. These are schematically illustrated in Figure 1, which displays from top to bottom the hole and electron transport between molecules (or fragments) A and B, the hopping of the first excited singlet state (S_1_) from A to B, and the coupling between an excited singlet on A and a singlet-coupled double triplet on A and a neighboring molecule (or fragment) B. The latter is known as singlet fission coupling. Singlet fission has the potential to significantly increase the efficiency of converting sunlight into electrical current since one photon can produce two electron–hole pairs.

The paper is organized as follows. After a short description of NOCI-F, Section 2 outlines the more approximate methods for the calculation of the electronic couplings. Section 3 introduces the organic molecules for which we calculated the electronic couplings for intermolecular transport and describes the computational information common to all calculations. The paper then continues with the description of the results, divided into four sections, one for each system. Most results are presented graphically, with the corresponding numerical values presented in the Supporting Information. The paper closes with a summary of the pros and cons of the approaches to calculate the couplings which can be used as a guideline to choose the method with an optimal trade-off between accuracy and computational effort in future studies of intermolecular energy and electron transport.

## 2 Computational Approaches

### 2.1 Nonorthogonal Configuration Interaction for Fragments

The matrix elements that appear in eq 3 are conveniently calculated with the nonorthogonal configuration interaction for fragments (NOCI-F) approach as implemented in GronOR.^1^ NOCI-F divides the system into fragments (for example, molecules A and B when looking at intermolecular energy or electron transport) and constructs many-electron basis functions (MEBFs) for the system from the wave functions that describe different electronic states of the fragments. The MEBFs are spin-adapted, antisymmetrized products of the fragments wave functions, which are typically expressed as linear combinations of Slater determinants to account for static electron correlation. Usually, the complete active space self-consistent field (CASSCF) approach is used to construct the fragment wave functions, but NOCI-F is flexible enough to accept any type of multiconfigurational wave function. Furthermore, the separate optimization of the orbitals for each fragment state ensures the full inclusion of orbital relaxation effects. Dynamic electron correlation can be included by shifting the diagonal matrix elements of the NOCI-F Hamiltonian or by effective Hamiltonian techniques.^2^ In both cases, the dynamic correlation between electrons situated on different fragments is neglected, which is a relatively small approximation given the short range of the dynamic electron correlation.

Expressing each fragment state in its own set of orbitals leads to mutually overlapping orbitals, and also the orbitals on different fragments are not necessarily orthogonal to each other. This makes the evaluation of the Hamiltonian and overlap matrix elements among the MEBFs more involved than in a standard approach with orbital orthogonality restrictions. However, the use of factorized transformed second-order cofactors^2,3^ and the massively parallel implementation in GronOR^1,4^ opened the door to perform NOCI calculations for systems with up to ≈150 atoms.

As can be inferred from Figure 1, the calculation of the coupling in the hole transport process involves fragment wave functions for the neutral ground state, labeled as *S*_0_, and the cationic doublet state, which we denote as D^+^. The MEBFs are then D^+^S_0_ for the initial state and S_0_D^+^ for the final state. After calculating the matrix elements , , , and ⟨D^+^S_0_|S_0_D^+^⟩, γ_if_^el^ is calculated by the substitution in eq 3. In the case of electron transport, the cationic state is replaced by the anionic doublet D^–^ and the corresponding MEBFs are D^–^S_0_ and S_0_D^–^. For the calculation of the coupling for exciton dispersion, the first excited singlet S_1_ enters into play and the MEBFs to be considered are S_1_S_0_ and S_0_S_1_. Finally, the singlet fission coupling requires the optimization of the following fragment wave functions: S_0_, S_1_, T_1_, D^+^, and D^–^. These fragment states are used to form the S_0_S_1_, S_1_S_0_, and T_1_T_1_ (the singlet coupled double triplet, schematically depicted in blue in Figure 1), and the charge transfer MEBFs D^+^D^–^ and D^–^D^+^. The latter are essential to include the effect of charge transfer configurations on the coupling, which is known to significantly enhance the coupling between the excited singlet and double triplet.^5,6^

### 2.2 Ab Initio Frenkel–Davydov

To reduce the computational cost of the calculation of the matrix elements in eq 3, Morrison, Zhou, and Herbert^7^ designed an ab initio implementation of the Frenkel–Davydov model.^8,9^ The ab initio Frenkel–Davydov (AIFD) approach is similar to NOCI-F in the basic aspects; it constructs MEBFs as antisymmetrized spin-adapted fragment wave functions and calculates the electronic couplings using the Hamiltonian and overlap matrix elements between nonorthogonal representations of the initial and final states. The main difference between AIFD and NOCI-F lies in the description of the fragment wave functions. AIFD aims at a favorable balance between accuracy and computational cost by using more approximate fragment wave functions than those typically used in NOCI-F. The S_0_ fragment wave function is approximated by a single Slater determinant, as are the D^+^ and D^–^ electronic states. For the description of the excited singlet state and the lowest triplet state (S_1_ and T_1_), configuration interaction of singles (CIS) is performed. That is, the wave function consists of the S_0_ reference determinant and all determinants that can be created by promoting one electron from an occupied orbital to an unoccupied orbital. CIS already leads to much shorter wave function expansions than the CASSCF approach typically applied in NOCI-F, but is even further reduced by transforming the orbitals to so-called natural transition orbitals (NTO).^10−12^ These NTOs are obtained from a singular value decomposition (SVD) of the one-particle transition density matrix 1TDM
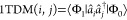
4where Φ_1_ is the CIS expansion of the S_1_ or T_1_ state, Φ_0_ is the ground-state wave function and  and  are the annihilation and creation operators for the occupied orbital *i* and the unoccupied orbital *j*. The SVD procedure leads to a set of corresponding orbitals^13^
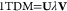
5where **U** and **V** are the hole and electron natural transition orbitals, and λ is a diagonal matrix with the singular values. The importance of the hole–electron pair *i* in the excited state is given by λ_*i*_. Re-expressing the excited state wave functions in the set of NTOs corresponding to the respective excitation leads to a very compact expansion that can become as small as two or three relevant contributions with minimal loss of accuracy when the hole–electron pairs with small λ values are neglected.

### 2.3 Frontier Molecular Approaches

Reducing the physics of an electronic transition of a system to the highest occupied molecular orbital (HOMO) and the lowest unoccupied molecular orbital (LUMO) leads to models that are attractive from a conceptual point of view. They have, in principle, the ability to provide additional understanding and explain tendencies when comparing different systems. The electronic states involved in the processes shown in Figure 1 can be expressed in a frontier molecular orbital model (FMO) containing the HOMOs and LUMOs of the two fragments. Smith and Michl derived the energy expressions and their interaction in terms of the one- and two-electron integrals of this four MO model assuming zero overlap among the orbitals,^5,14^ which were later generalized for overlapping orbitals by Buchanan et al.^15^ To enable a fast exploration of the relative orientation of the dimers and an efficient screening of different materials for singlet-fission properties, additional approximations were introduced such as the use of minimal atomic basis sets and zero differential overlap.^15,16^

Here we compare the outcomes of the NOCI-F calculations with the estimates based on the following four-orbital FMO model. The orbitals of the ground state of both fragments are optimized in two standard Hartree–Fock calculations and subsequently superimposed in the appropriate dimer geometry. The HOMO and LUMO of both fragments are Löwdin orthogonalized^17^ and the energies (*H*_*ii*_) and interactions (*H*_*ij*_) of the states are determined by the equations given in the Supporting Information. The coupling γ_if_^el^ is equal to the *H*_*if*_ because of the orthogonality of the states.

The second HOMO–LUMO model that is tested is the so-called Dimer Projection (DIPRO) approach, proposed by Baumeier, Kirkpatrick, and Andrienko^18^ to study intermolecular charge transfer with density functional theory. This approach projects the Kohn–Sham (KS) or Hartree–Fock (HF) orbitals of the molecules A and B on the basis of the dimer AB and then applies a simplified version of eq 3
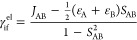
6to estimate the coupling for the charge transport processes. The coupling for hole transport involves the HOMOs of the two molecules, and for electron transport, one should use the two LUMOs. In this equation, *S*_AB_ stands for the overlap of the HOMOs or LUMOs of the two fragments, ϵ_A_ and ϵ_B_ are the corresponding orbital energies and *J*_AB_ is given by
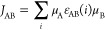
where *i* runs over all occupied orbitals of the dimer and ε_AB_(*i*) is the orbital energy. μ_A_ and μ_B_ are the projections of the monomer orbitals onto the dimer.

### 2.4 Effective Hamiltonian Techniques

Another approach for obtaining estimates of the coupling between different electronic states is the construction of an effective Hamiltonian. A collection of adiabatic states of the system under study is projected onto a model space  spanned by the diabatic states for which the coupling needs to be calculated. The projected states are orthogonalized (Ψ^⊥^) and the Bloch formula
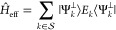
7is used to construct the effective Hamiltonian. The off-diagonal elements of  are measures of the coupling between the diabatic states.

To illustrate the approach, we briefly discuss the calculation of the coupling between two singlet excitons localized on fragments A and B. The first step involves a state average CASSCF (SA-CASSCF) calculation on the combined A–B system, ensuring that the calculation includes the states dominated by the electronic configuration representing the local excited singlets. The active orbitals are typically delocalized over the two fragments, which makes it difficult to identify the excited singlet configurations and obstructs the construction of the effective Hamiltonian. Therefore, the active orbitals are localized by projecting a set of model vectors in the active space based on the approach described in ref (19). The SA-CASSCF wave function is re-expressed in the localized orbitals and the electronic states with the largest projection on the model space  are selected. These are not necessarily the lowest two excited states, because other states, for example, the singlet-coupled double triplet state, may have lower energies. Finally, the coefficients of the orthogonalized projections and the SA-CASSCF energies of the roots with the largest projection are used to construct the effective Hamiltonian.

When the two fragments of the system are related to each other by inversion symmetry, the two singlet excitons appear as gerade and ungerade combinations of the left- and right-localized excitons in the SA-CASSCF. In that case, there is no need for an effective Hamiltonian as the coupling is equal to the energy difference of the states divided by two.^20^

### 2.5 Transition Dipole Moment-Based Couplings

The next method that is compared to NOCI-F is the transition dipole moment coupling (TDC) model of Abe, Moore, and Krimm.^21,22^ The method has been used primarily to describe infrared and Raman spectra of proteins in the amide region^23−26^ but is more generally applicable.^27^ TDC relates the coupling of initial and final states to the scalar product of the transition dipole vectors between ground and excited states on the two molecules
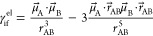
8where  and  are the transition dipole vectors of the S_0_ → S_1_ excitation on A and B, and r_AB_ is the distance between the centers of mass of the two molecules. TDC is part of the classical Förster theory for energy transfer^28^ and taking the square of eq 8 leads to the well-known 1/*r*^6^ dependency of the transfer rate of the Förster resonance energy transfer (FRET). The comparison with NOCI-F is restricted to exciton dispersion only since this is the only process where dipole–dipole interactions are relevant. The other processes either involve spin-forbidden local transition (singlet fission coupling) or change the number of electrons on the monomers (hole and electron transport).

### 2.6 Property-Based Diabatization

Except for the method based on effective Hamiltonians, the methods discussed above are based on diabatic representations of the initial and final states constructed by superimposing the wave functions of the separate fragments. However, this is not the only possible way of representing diabatic states, and in fact, the magnitude of the electronic coupling depends on the details of the diabatization scheme. Among the many different diabatization schemes,^29−31^ we have selected the Mulliken–Hush approach^32,33^ to explore the dependency of the calculated electronic coupling on the representation of the diabatic states. The Mulliken–Hush approach uses the dipole moment operator to define diabatic states and the resulting electronic coupling can be expressed in properties of the adiabatic initial and final states as
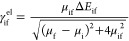
9where μ_if_ is the transition dipole moment and μ_i_ and μ_f_ are the dipole moments of the initial and final states. Note that the expression reduces to  when the dipole moments of the initial and final adiabatic states are equal or zero.

## 3 Computational Information

**Figure 2:**
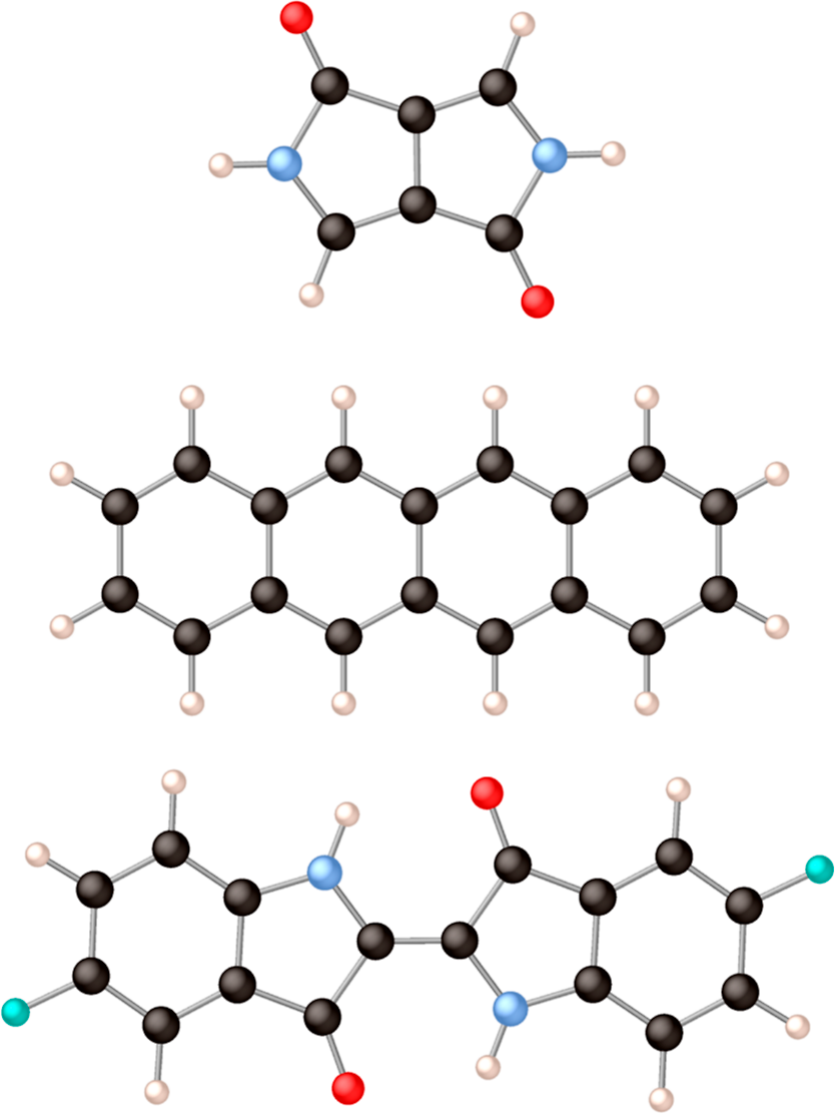
From top to bottom: diketopyrrolopyrol (dpp), tetracene, and 5,5′-difluoroindigo. Black spheres represent carbon atoms, light blue is nitrogen, red is oxygen, white is hydrogen, and mint-green spheres are fluorine atoms.

Figures 2 and 4 depict the molecules that were studied to compare the performances of the different computational schemes to calculate the electronic couplings. The study starts with diketopyrrolopyrol (dpp), which is a simple model system for larger molecules with singlet fission properties or that can act as organic conductors. The second molecule, tetracene, is a member of the acene family that has been intensively studied for its singlet fission properties. Next, we focus attention on the 5,5′-difluoroindigo molecule, which has been found to be an organic conducting material^34^ and also suggested to be an interesting candidate to act as photosensitizer for dye-sensitized solar cells.^35^

**Figure 3:**
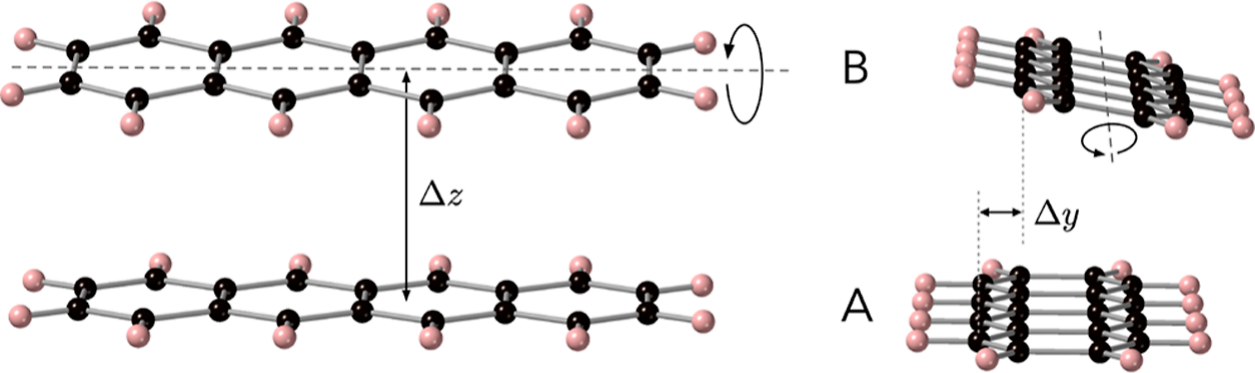
Relative orientation of the tetracene molecules A and B and definition of the rotation axis for molecule B.

**Figure 4:**
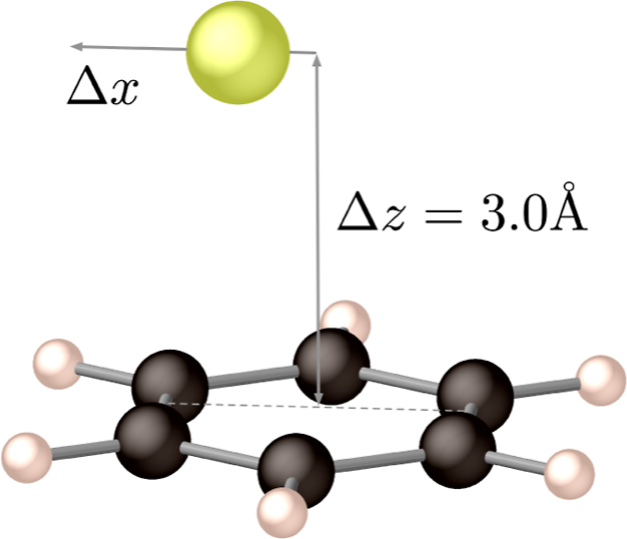
Benzene–Cl complex with the chlorine atom (in yellow) at 3.0 Å above the molecular plane of benzene and at variable displacement along the *x*-axis.

In the case of 5,5′-difluoroindigo, the geometry of the dimers used to calculate the electronic couplings was taken from experiment. Two different pairs of molecules can be recognized in the crystal structure of this compound. In the first place, there are the parallel molecules forming stacks, and second, pairs of molecules from neighboring stacks, whose molecular planes show an angle of approximately 67° as will be shown in Section 6. For dpp and tetracene, pairs of molecules have been considered to be not taken from the experimental structure but rather exploring the relative orientation of the two molecules in a series of calculations in which the intermolecular distance is steadily increased or one of the molecules is rotated around the internal molecular axis as shown in Figure 3 for tetracene.

The systems described above are not adequate for a Mulliken–Hush evaluation of the electronic coupling because the dipole moment of initial and final adiabatic states are strictly the same. For this reason, the coupling for electron transport in the benzene–Cl system was studied, as previously done by Cave and Newton.^32^

Valence double-ζ plus polarization one-electron basis sets taken from the ANO-S library of the OpenMolcas program suite^36^ have been used in all calculations. The multiconfigurational wave functions used in the NOCI-F calculations are constructed through the CASSCF approach with different active spaces. The size of the active space and the nature of the active orbitals are specified in the discussion of the results for each particular system. The thresholds, τ_MO_ and τ_CI_, controlling the size of the common molecular orbital basis and the number of determinant pairs considered in the evaluation of the nonorthogonal matrix elements, are 10^–4^ and 10^–5^ showing the optimal balance between accuracy and computational effort.^1,37^

The CIS calculations used to describe the S_1_ and T_1_ states in the AIFD approach were done by allowing single excitations from the *n* occupied π orbitals of the molecule under study to the 75-*n* lowest virtual orbitals. The threshold for keeping single excitations after the transformation to NTOs, the singular value λ, was set to zero.

The definition of the density functionals has been taken from the LIBXC library.^38^ Automatically generated atomic Cholesky decomposition auxiliary basis sets were used to treat the two-electron integrals.^39^

## 4 Results: Diketopyrrolopyrol (dpp)

### 4.1 Hole and Electron Transport

**Figure 5:**
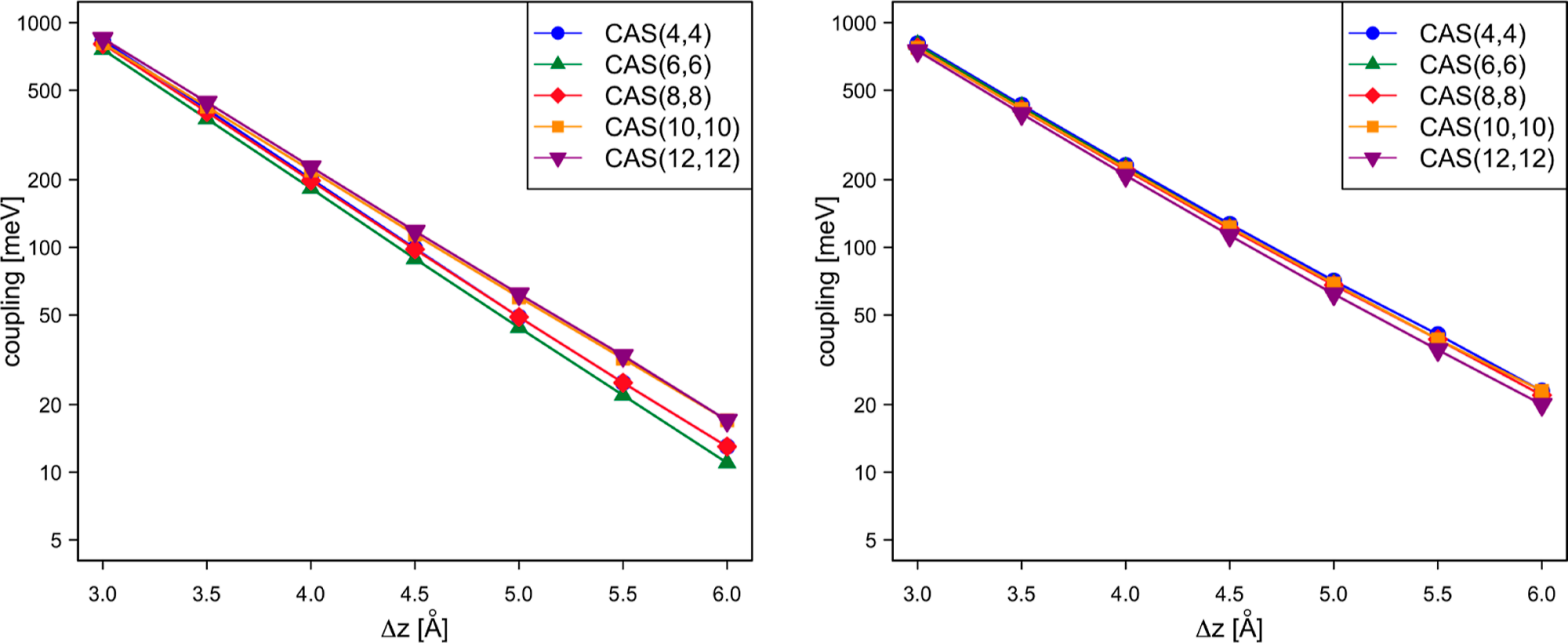
NOCI-F electronic couplings (in meV) for hole transport (left) and electron transport (right) as a function of the intermolecular distance of two perfectly stacked parallel dpp molecules for different active spaces for the fragment wave functions.

While earlier NOCI-F studies addressed the dependence of the results on the size of the common molecular orbital basis and the threshold for considering determinant pairs in the evaluation of the matrix elements,^1,2,37^ less attention has been paid to the influence of the size of the active space used to construct the fragment wave functions. Starting with the electron and hole transport in dpp as a function of the intermolecular distance between two perfectly stacked parallel molecules, Figure 5 illustrates the dependence of the coupling calculated with NOCI-F as a function of the size of CAS. The figure clearly shows that the size of the active space has limited influence on the calculated coupling in both cases. There is a slight tendency toward larger couplings with increasing active space in the case of hole transport, but the differences are never substantial. The labels used to discern the different active spaces refer to the S_0_ fragment state. For the D^+^ and D^–^ states, the CAS counts with the same number of orbitals, but with one electron less (D^+^) or one electron more (D^–^) than indicated by the label. The active orbitals of the CAS(12,12) are graphically represented in Figure S1 of the Supporting Information and Tables S1 and S2 contain the numeric values of the couplings.

**Figure 6:**
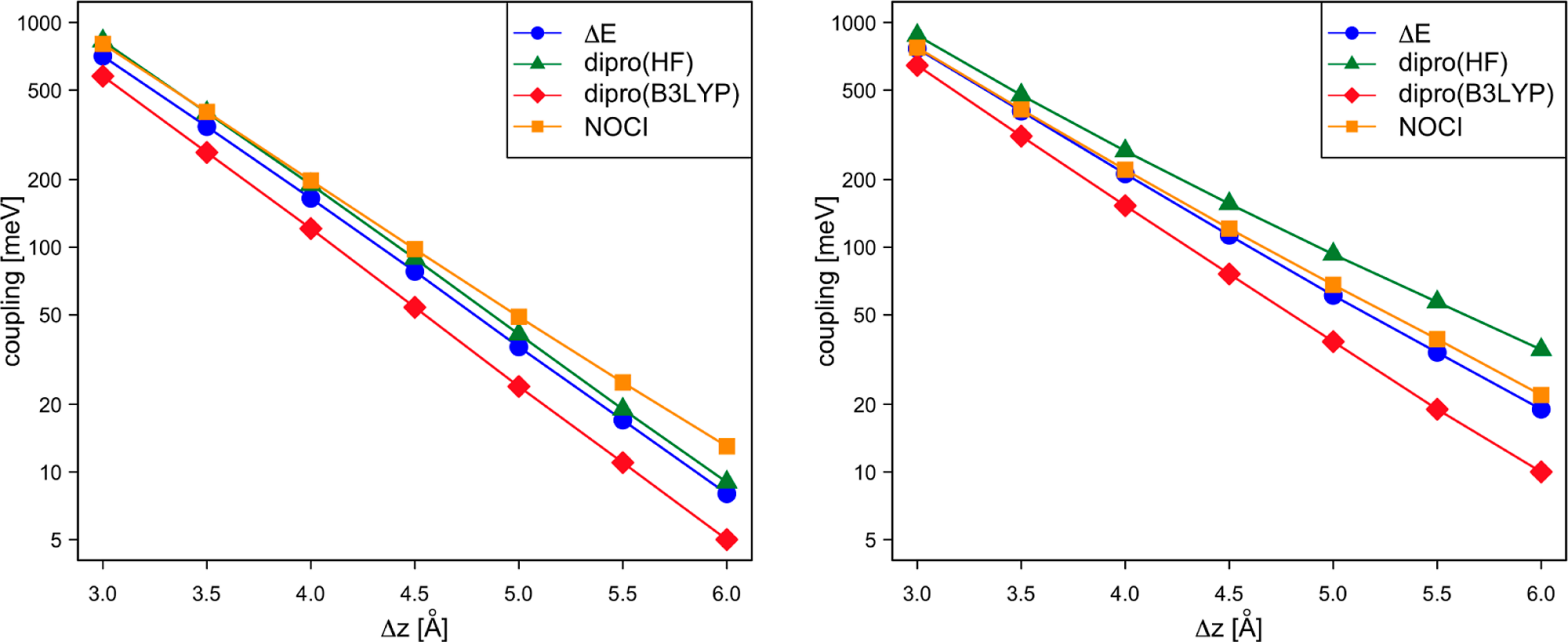
Electronic coupling (in meV) for hole transport (left) and electron transport (right) as a function of the intermolecular distance of two perfectly stacked parallel dpp molecules.

Figure 6 compares the electronic coupling calculated with NOCI-F based on CAS(8,8) fragment functions, the DIPRO approach using HF and B3LYP KS orbitals, and the results extracted from the energy difference of the gerade and ungerade states of the dimer (Δ*E*, see Section 2.4). The dimer calculations were done with an active space of 16 orbitals and 15 or 17 electrons, the same number of orbitals and electrons used to express the S_0_D^±^ and D^±^S_0_ MEBFs in the NOCI-F calculations. It is observed that NOCI-F and the Δ*E* calculations give very similar results. DIPRO with B3LYP orbitals slightly underestimates the coupling in both cases, while the use of HF orbitals leads to couplings that are nearly identical to the NOCI-F results for hole transport and somewhat larger for electron transport.

The observation that the DIPRO couplings show a certain dependence on the orbitals has been further elaborated by repeating the calculations with a collection of density functionals of different types: LDA, GGA, *meta*-GGA, and hybrid functionals. The spread in the calculated values is substantial (see Supporting Information, Tables S3, S4, and Figure S2) and to obtain further insight into the origin of this spread, we have performed a series of DIPRO calculations with the density functional α[ρ_*x*_^HF^] + (1 – α)[ρ_*x*_^B88^] + β[ρ_C_^LYP^], varying α between 0.9 and 0.1 and β equal to 1 or 0. α controls the amount of exact Fock exchange in the functional, while for β = 0 the correlation part of the functional is completely switched off. The results in Table 1 show that the couplings systematically decrease when the amount of exact Fock exchange diminishes but are insensitive to the deactivation of the correlation part of the functional. The B3LYP values at 3 Å reported in Figure 6 are very close to the values calculated here with α = 0.2, the amount of exact Fock exchange in the B3LYP functional. Computational estimates of the electronic coupling for hole transport based on energy differences of dimer states performed for transition metal oxides^40^ point into the same direction: these parameters are not strongly dependent on the inclusion of dynamic correlation in the computational treatment. The steady decrease of the coupling with decreasing exact Fock exchange suggests that the residual self-interaction inherent to DFT^41^ plays a role in the tendency, although one should realize that quantifying the self-interaction error is far from trivial for multielectronic systems.^42^ The self-interaction leads to slightly more delocalized orbitals and affects the overlap (S_AB_), orbital energies (ε_A,B_), and also the projection onto the dimer basis (*J*_AB_), see Supporting Information, Table S5. While the tendency of the coupling for electron transport is clearly dominated by the changes in the orbital energies, the trend in the coupling for hole transport is more complex (see Supporting Information, Figure S3). Especially, the substantial changes in *J*_AB_ make it complicated to establish a one-to-one relation with the self-interaction error.

**Table 1:** DIPRO Couplings (in meV) for Hole and Electron Transport for Two Perfectly Stacked Parallel dpp Molecules (Δ*z* = 3.0 Å) by Applying the Hybrid Functional α[ρ_**x**_^HF^] + (1 – α)[ρ_**x**_^**B**88^] + β[ρ_**C**_^LYP^]

	β = 1		β = 0	
α	hole	electron	hole	electron
0.9	796	883	794	857
0.8	763	849	761	831
0.7	731	815	728	802
0.6	699	781	696	770
0.5	667	746	665	738
0.4	637	712	634	706
0.3	606	678	604	673
0.2	577	645	574	640
0.1	549	612	546	608

### 4.2 Exciton Transfer

**Figure 7:**
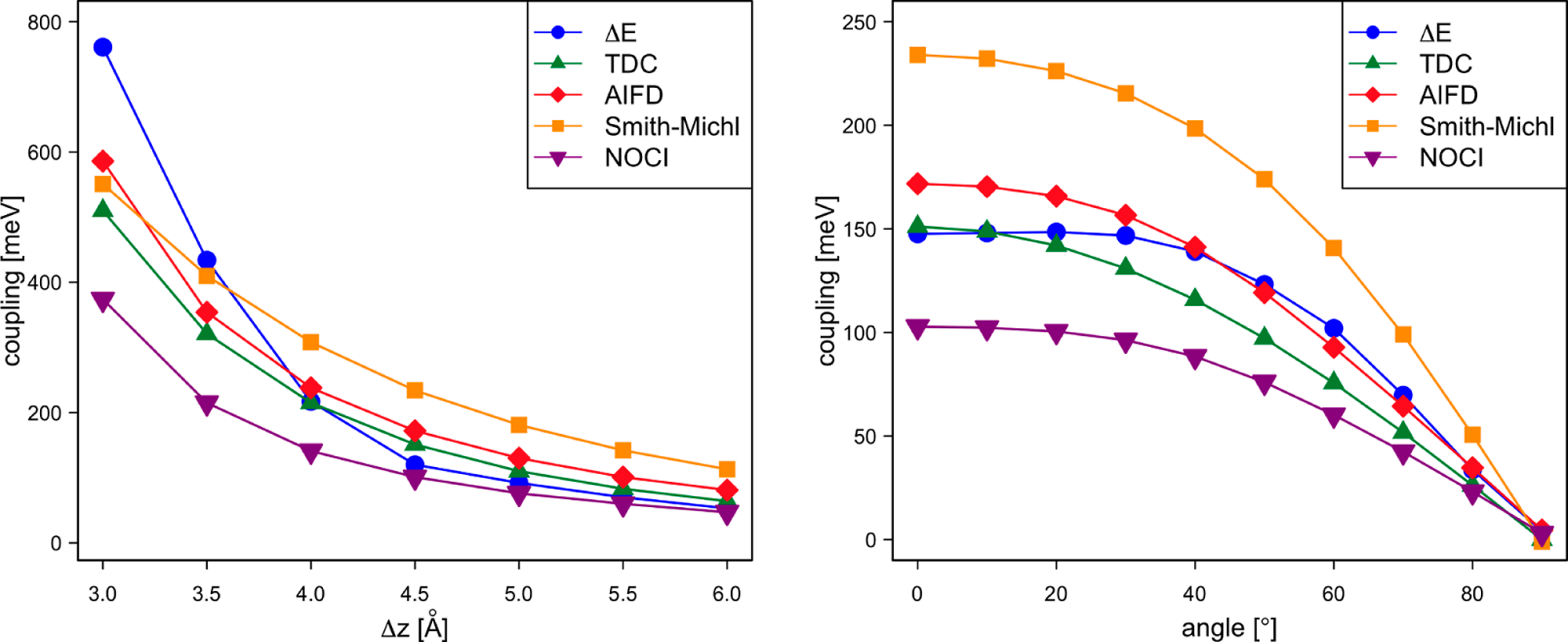
Electronic coupling (in meV) for exciton transport as a function of the intermolecular distance of two perfectly stacked parallel dpp molecules (left) and as of function of the rotation angle of the second dpp molecule situated at Δ*z* = 4.5 Å (right).

The performance of the computational schemes outlined in Section 2 for intermolecular energy transfer is first addressed by the calculation of the transfer of a local excited singlet state from one dpp molecule to a neighboring one. The left part of Figure 7 compares the different estimates for two perfectly stacked dpp molecules at different intermolecular distances. This coupling is not defined within the DIPRO approach, but instead, it can be estimated with the Smith–Michl model and from the local transition dipole moment couplings (TDC). We have also added the estimates extracted from the ab initio Frenkel–Davydov (AIFD) approach. The strength of the coupling is comparable to the couplings discussed in the previous section, but the differences between the approaches are significantly larger than for the hole/electron transport. Compared with couplings estimated with NOCI-F using CASSCF(8,8) fragment wave functions, the other methods predict stronger couplings. The couplings derived from the energy differences of the CASSCF(16,16) dimer states (Δ*E*) are very close to the NOCI-F results at the larger distances but strongly deviate at the shorter intermolecular distances. The other approaches rather accurately follow the tendency of the NOCI-F calculations. This is especially remarkable for TDC, given that this approach is typically applied when the distance between the chromophores is much larger.

The dependency of the couplings on the size of the active space is reported in the Supporting Information (Tables S6 and S7). The NOCI-F couplings remain practically unchanged once the active space for the fragment wave function has increased up to at least eight electrons and eight orbitals, although the results with the smaller active spaces are not too different either. The estimates based on the CASSCF calculations on the dimer (Δ*E*) are also not strongly dependent on the size of the active space, except for the smallest intermolecular distance (see Table S7). The TDC coupling and the transition dipole moment itself are only weakly dependent on the choice of the CAS, and their dependency on the one-electron basis set size is also minor, as reported in Tables S8, S9, and S12.

A similar picture arises by comparing the electronic couplings when one of the molecules is rotated along the molecular axis, as shown in Figure 3. Again NOCI-F predicts the smallest couplings, and the other methods reproduce the same tendency, albeit with larger couplings (Figure 7, right). TDC remains closest to the NOCI-F values. Note that in the present setup where the second molecule is only displaced along the *z*-axis, the second term in eq 8 is strictly zero because  is orthogonal to  for all geometries. To address the importance of this second term, we have repeated the couplings reported in Figure 7 translating molecule B by 1.0 Å along *x* and *y*. The results are reported in the Supporting Information (Tables S10, S11 and Figures S4, S5) and show that the second term gives a non-negligible contribution, especially for the larger rotation angles.

To obtain the Δ*E* estimates the full procedure described in Section 2.4 has to be applied because the dimer loses its inversion symmetry for nonzero rotation angles. Actually, the CASSCF calculations in themselves are also significantly more complicated due to the lack of symmetry in the dimer. The two electronic states required to extract the coupling are no longer the lowest roots in the CASSCF calculation but are in fact extracted from a state-average CASSCF with six electronic states. Moreover, these states are not easily identifiable as the plus and minus linear combination of the local excited singlets (S_0_S_1_ ± S_1_S_0_), which makes the norm of the projection on the model space smaller, especially for the larger rotation angles where the electronic states become nearly degenerate. Finally, state-average CASSCF with six roots makes the use of an active space with 16 electrons and 16 orbitals computationally very expensive. The Δ*E* results represented in the right panel of Figure 7 are obtained with a CAS(8,8). Given the modest dependency of the couplings on the size of the active space as a function of the intermolecular distance (see Table S7 of the Supporting Information), we do not expect very large changes here and assume that the CAS(8,8) results are representative. It would be very interesting to compare the Δ*E* results to those obtained with localized active space state interaction calculations.^43^ This is the subject of ongoing research.

### 4.3 Singlet Fission Coupling

The second example of intermolecular transfer concerns the coupling between a local excited singlet state and the singlet coupled double triplet state, as schematically represented in the last entry of Figure 1. This so-called singlet fission coupling can be estimated by considering only the local singlet and triplet states, giving rise to the so-called direct coupling. However, the strength of the direct coupling is known to underestimate the efficiency of the energy transfer and a more accurate description of the process is obtained when one also considers the charge-transfer states.^5,6,44^

**Figure 8:**
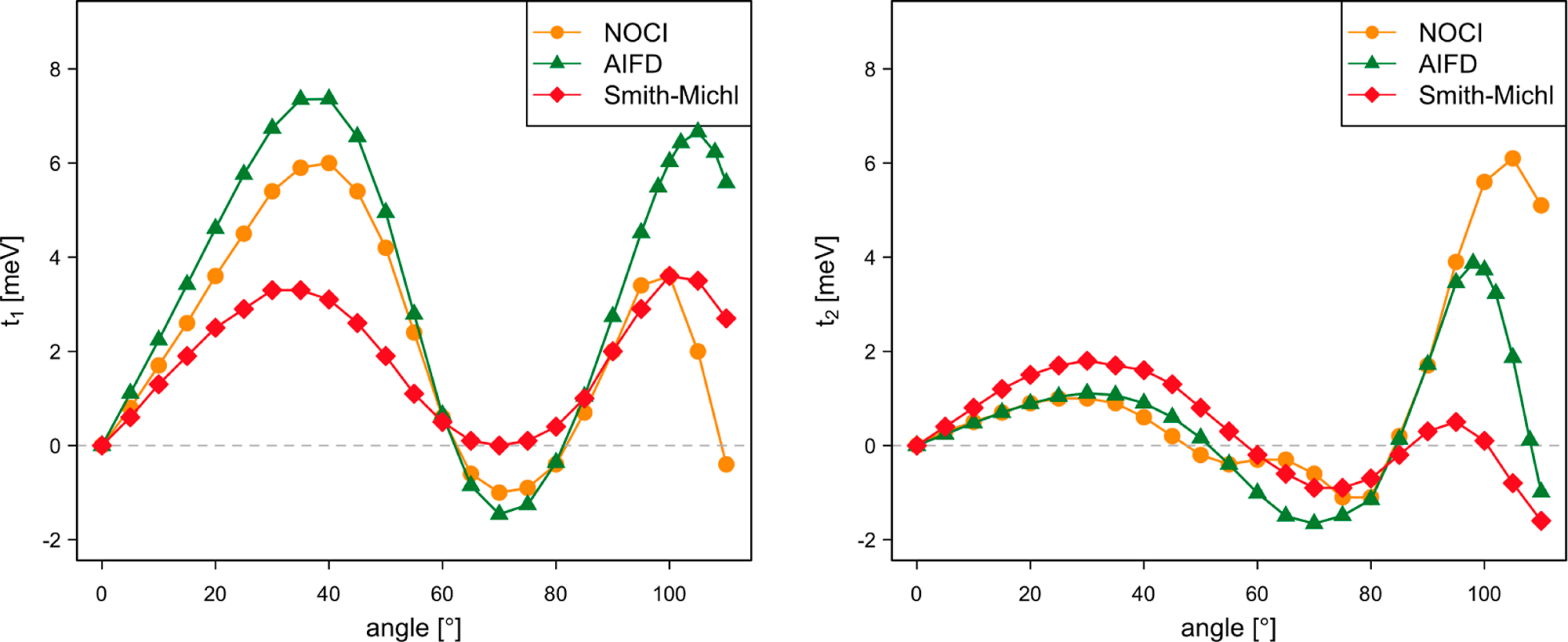
Direct singlet fission couplings *t*_1_ and *t*_2_ (in meV) as a function of the rotation angle of the molecular plane of the second dpp situated at Δ*z* = 4.5 Å.

The values displayed in Figure 8 correspond to the coupling of the *c*_1_·S_0_S_1_ ± *c*_2_·S_1_S_0_ state with the ^1^TT state, where the minus combination of local singlets is used to calculate *t*_1_ (left panel) and the plus combination gives rise to *t*_2_ (right panel). The NOCI-F results are obtained with CASSCF(8,8) fragment wave functions, and the effect of the size of the CAS on the coupling is limited (Figure S6). For small rotation angles, the absolute values of *c*_1_ and *c*_2_ are almost equal, but they gradually evolve toward 0 and 1, respectively, with increasing angle (Figure S7). The comparison of the NOCI-F couplings to those calculated with the more approximate AIFD and Smith-Michl approaches shows that the latter reasonably well reproduce the NOCI-F values. The only exceptions are the Smith–Michl *t*_2_-values for large angles, which remain close to zero while the other two methods predict a substantial increase of the coupling.

**Figure 9:**
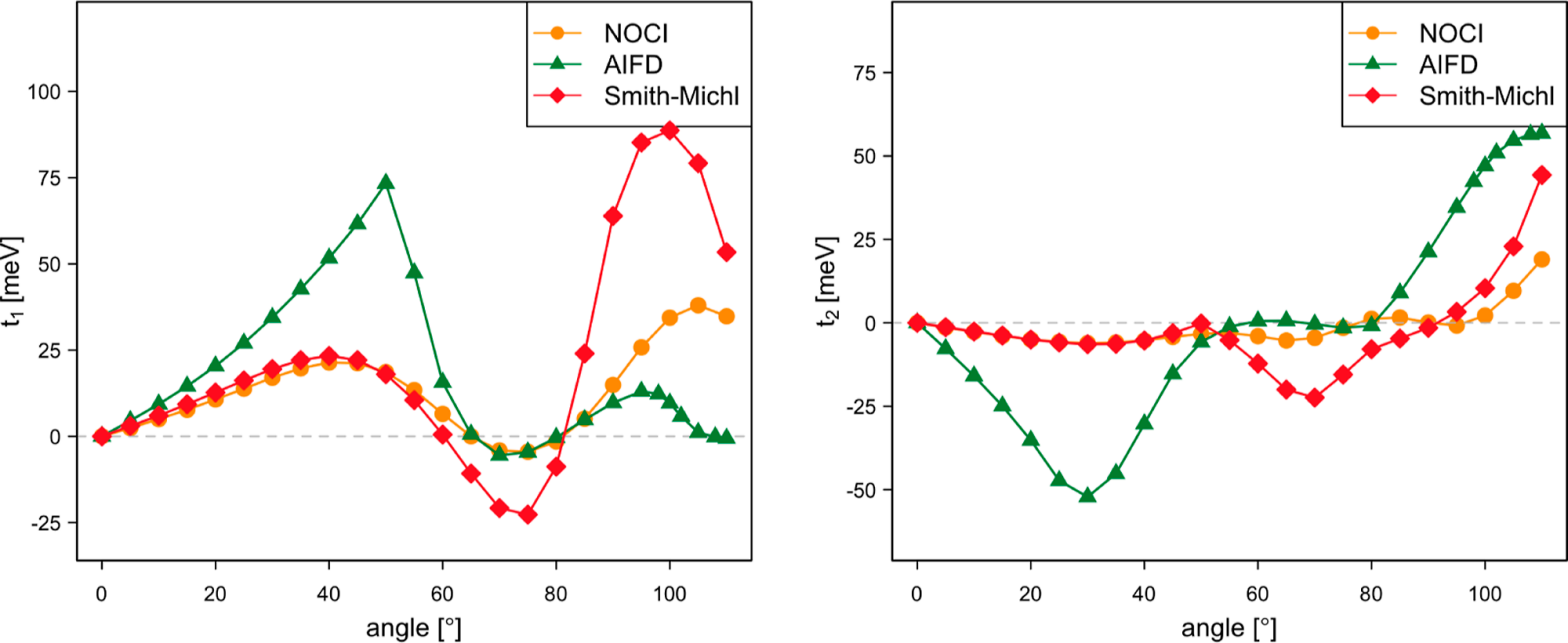
Total singlet fission couplings *t*_1_ and *t*_2_ (in meV) as a function of the rotation angle of the molecular plane of the second dpp situated at Δ*z* = 4.5 Å.

**Figure 10:**
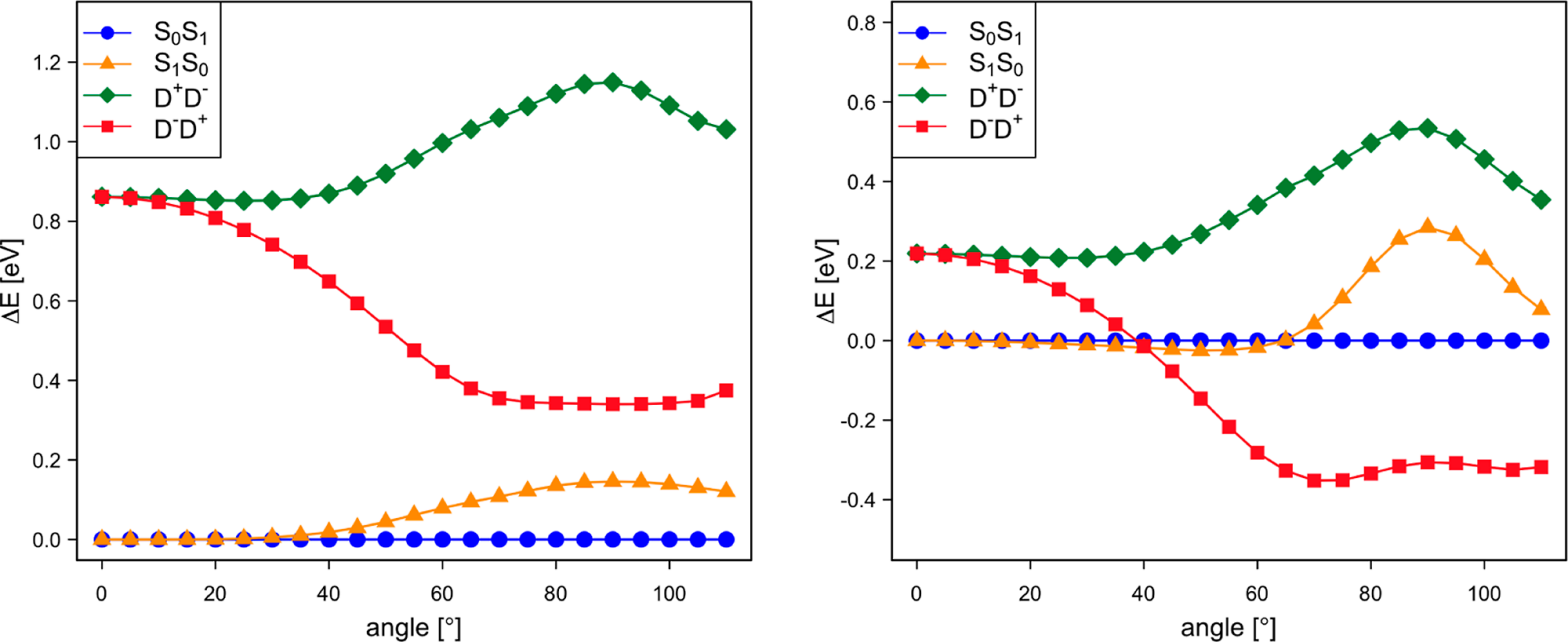
NOCI-F (left) and AIFD (right) relative energies (in eV) of the exciton and CT MEBFs as a function of the rotation angle of the molecular plane of the second dpp situated at Δ*z* = 4.5 Å.

As expected, the NOCI-F coupling is significantly enhanced when the effect of the charge transfer (CT) states is taken into account, as can be seen in Figure 9. This is most obvious for *t*_1_, but the smaller *t*_2_ coupling is also significantly larger than the direct one, especially for large rotation angles. This total coupling is also relatively insensitive to the size of the CAS used to calculate the fragment wave functions. The largest deviations are observed for the NOCI-F based on CAS(4,4) MEBFs (see Figure S8). However, the comparison with the other two methods is less favorable when CT is included. The Smith–Michl performs quite well for angles up to 45° but overestimates the coupling at the larger angles. The AIFD approach gives poor results and seems to be unfit to predict couplings with the CT effects incorporated. The origin of these apparently wrong results is directly related to the energies of the D^+^D^–^, D^–^D^+^ MEBFs with respect to those that are used to describe the excitonic states (S_0_S_1_ and S_1_S_0_). As shown in Figure 10, AIFD places the CT states about 0.6 eV lower in energy than that in the NOCI-F for all rotation angles. Consequently, the effect of the CT states in the coupling is largely overestimated in the AIFD calculations, and for rotation angles larger than 45°, the results become unreliable as one of the CT MEBFs becomes more stable than the local singlet states.

**Figure 11:**
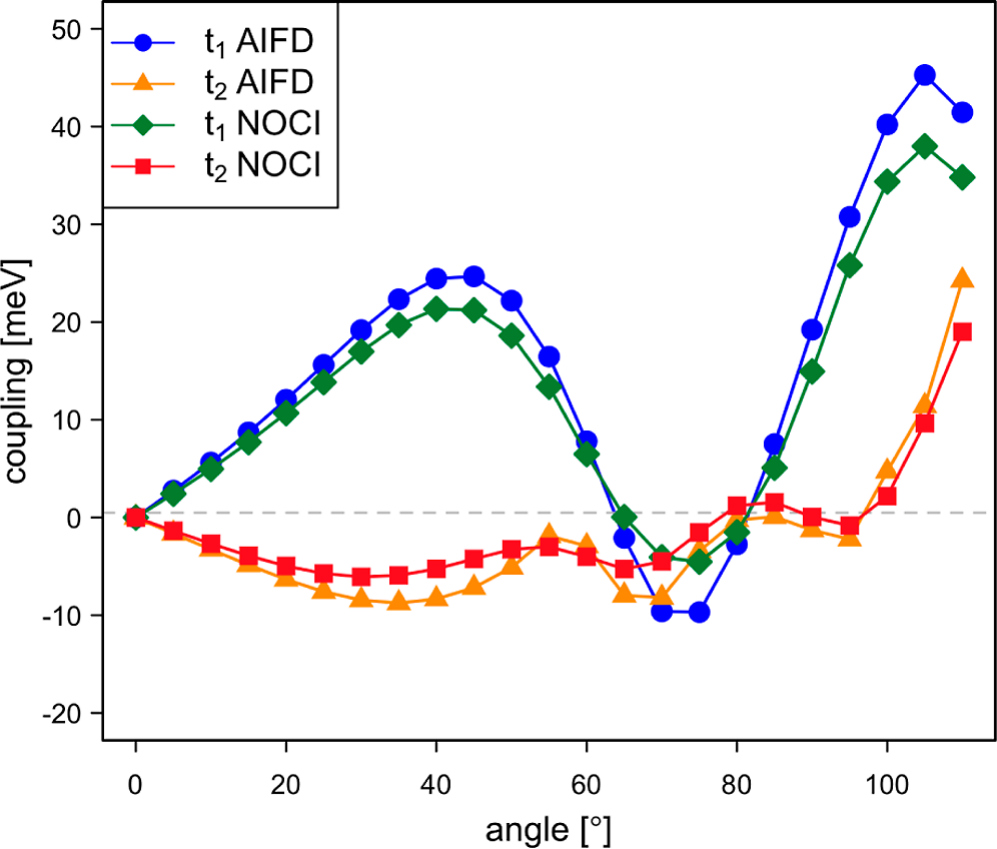
NOCI-F and shifted AIFD singlet fission couplings *t*_1_ and *t*_2_ (in meV) as a function of the rotation angle of the molecular plane of the second dpp situated at Δ*z* = 4.5 Å.

To check that the poor AIFD results are indeed caused by the too low energies of the CT MEBFs, we have performed a new series of AIFD calculations in which the CT MEBFs were uniformly shifted to higher energy by 0.6 eV in the whole interval of angles. In practice, this can be done by modifying the diagonal matrix elements of the Hamiltonian as described in ref (2). Applying the shift leads to a spectacular improvement in the AIFD couplings; both *t*_1_ and *t*_2_ now closely follow the NOCI-F values, see Figure 11.

## 5 Results: Tetracene

### 5.1 Hole and Electron Transport

**Figure 12:**
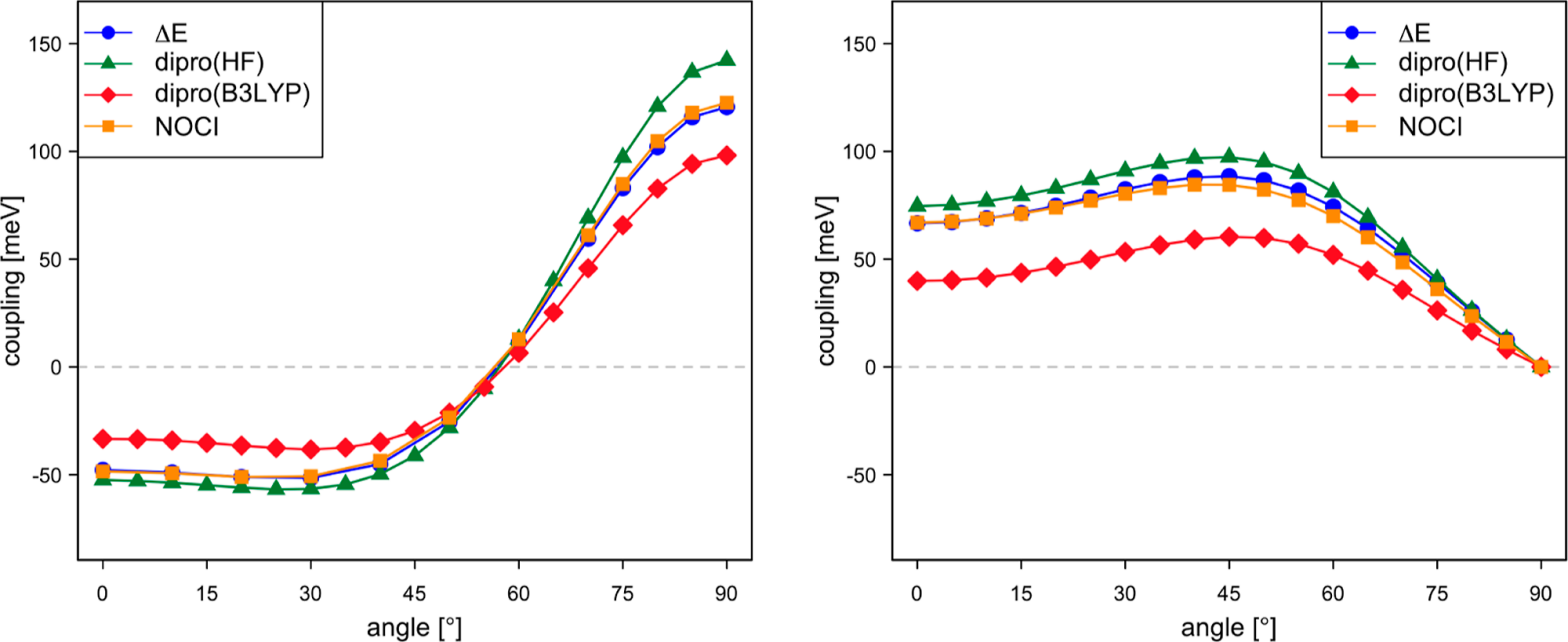
Electronic coupling for hole (left) and electron transport (right) as a function of the rotation angle of tetracene B.

The dependency of electronic couplings for hole and electron transport on the intermolecular distance between two perfectly stacked tetracene molecules is similar to what was observed for dpp and is reported in the Supporting Information (Figure S9). Here, we discuss in brief how these couplings vary when one of the molecules in the dimer is rotated, as shown in Figure 3, and to what extent the different approximate approaches are capable of following the NOCI-F results. The NOCI-F fragment wave functions are calculated with an active space of 10 electrons in 10 orbitals, see Figure S10 for a graphical representation of the active orbitals of the S_1_ fragment state. Figure 12 shows how the couplings for hole transport are nearly constant for angles up to 35° then rapidly increase, change sign at approximately 60°, and continue to grow to reach a maximum at 90°. Note that the sign of the coupling itself is not very relevant, as it depends on the sign of the wave functions of initial and final states. The coupling enters squared in Fermi’s golden rule (eq 2). The coupling for electron transport behaves differently, it is large for small angles, goes through a shallow maximum around 45°, and rapidly decays to zero at 90°. It is observed that the Δ*E*-based results are nearly indistinguishable from the NOCI-F values. The DIPRO couplings also closely follow the NOCI-F trends, with those based on HF orbitals predicting slightly stronger couplings and those with B3LYP KS orbitals giving rise to weaker couplings.

### 5.2 Exciton Transfer

**Figure 13:**
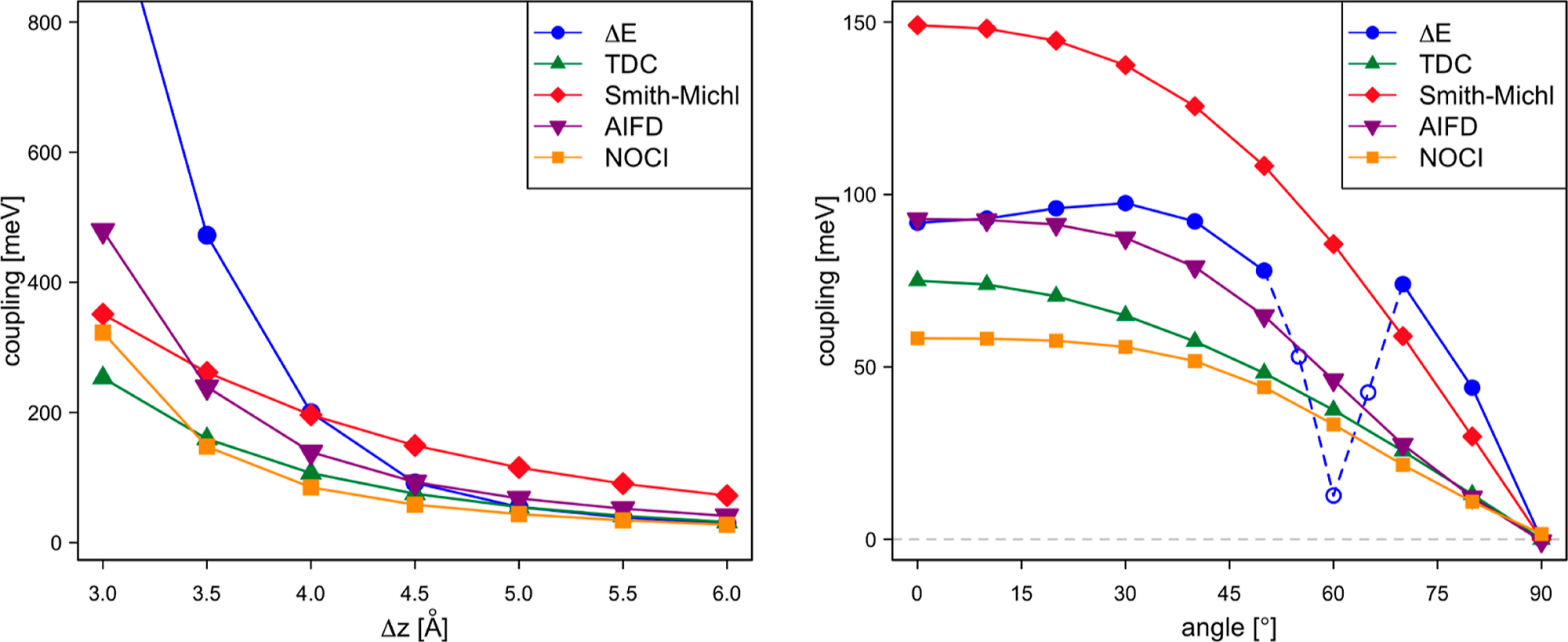
Electronic coupling for exciton transport as a function of Δ*z* (left) and the rotation angle of tetracene B (right).

The coupling for exciton transfer between two parallel tetracene molecules steadily decreases with an increasing Δ*z*, as reproduced by all methods. Figure 13 shows that TCD, AIFD, and Smith–Michl stay relatively close to the NOCI-F prediction for all intermolecular distances. As found for the dpp chromophore, the TDC approach leads to couplings that are closest to the NOCI-F values. The estimates extracted from SA-CASSCF calculations on the dimer (Δ*E*) strongly overestimate the coupling at short distances. The spread in the couplings is larger when rotating one of the tetracenes around its internal axis, maintaining the Δ*z* constant at 4.5 Å. The Smith–Michl model predicts couplings that are a factor of 2.5 larger than the NOCI-F couplings, the AIFD values are approximately 1.5 times larger. Whereas TDC, AIFD, and Smith–Michl follow the same tendency as NOCI-F, the couplings based on the SA-CASSCF dimer calculations not only overestimate these but also show a remarkable irregularity at 60° rotation. The reason is the following: For reliable estimates of the exciton coupling, the projections of the SA-CASSCF states on the model space should be sizable. When the two tetracenes are parallel, the S_1_S_0_ ± S_0_S_1_ electronic states are well separated from the other states, and the projections on the model space are large, ∼0.87 for both states. The separation between the electronic states remains relatively constant up to rotation angles of 45° (see Figure S11), and then rapidly decreases. This leads to electronic states that become strong mixtures of different electronic configurations, and at a rotation of 60°, the two states with the largest S_1_S_0_ ± S_0_S_1_ character have projections of 0.70 and 0.26 on the model space. This is obviously too low to obtain reliable answers. The situation improves for larger rotations: at 80°, the projections increase to 0.88 and 0.65. This makes the Δ*E*-based couplings more reliable again for larger rotations.

### 5.3 Singlet Fission Coupling

**Figure 14:**
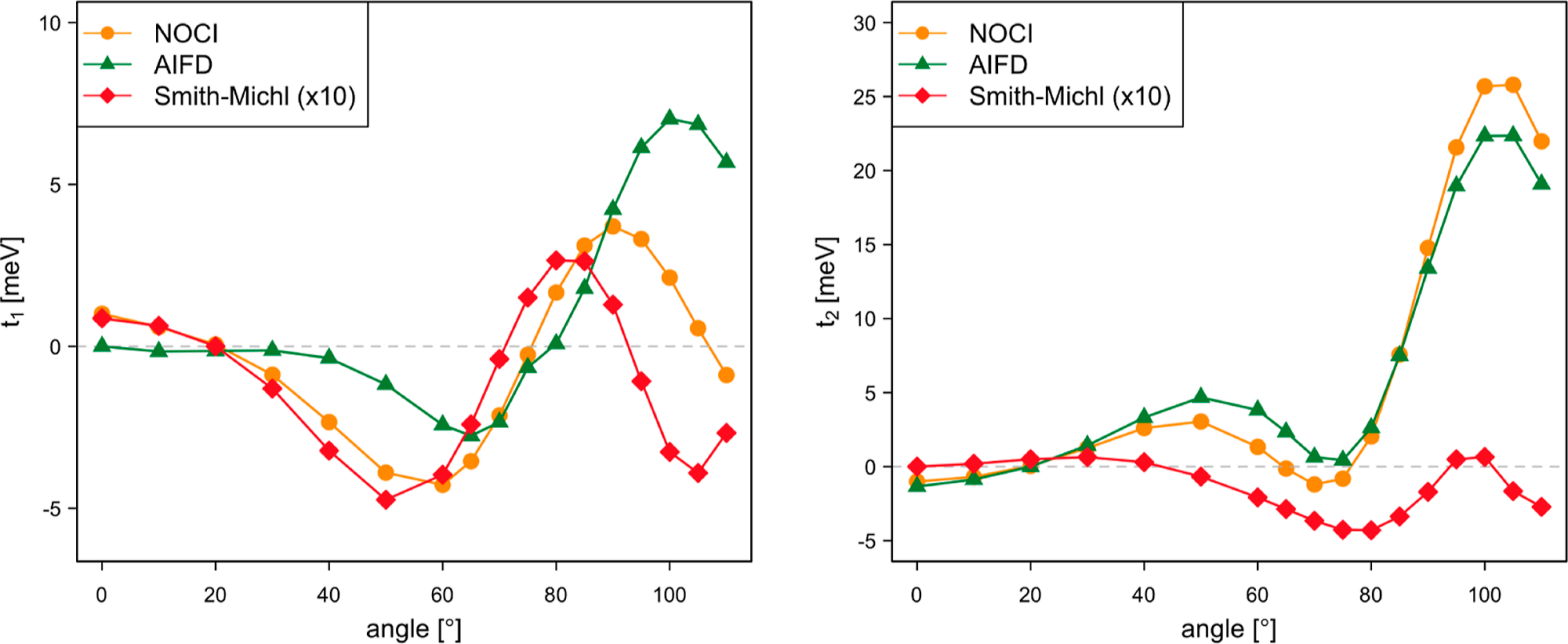
Singlet fission direct couplings *t*_1_ and *t*_2_ (in meV) as a function of the rotation angle of tetracene B.

The singlet fission couplings are calculated as a function of the rotation angle with Δ*y* = 0.75 Å and Δ*z* = 4.25 Å (see Figure 3). The NOCI-F direct SF couplings depicted in Figure 14 are small for small rotation angles, show a minimum (*t*_1_) or maximum (*t*_2_) around 45°, and go through a maximum at rotation angles close to 90°. The second maximum is particularly pronounced for *t*_2_. AIFD reproduces the NOCI-F trends, but the direct SF couplings arising from the expressions of the Smith–Michl model are an order of magnitude too small.

**Figure 15:**
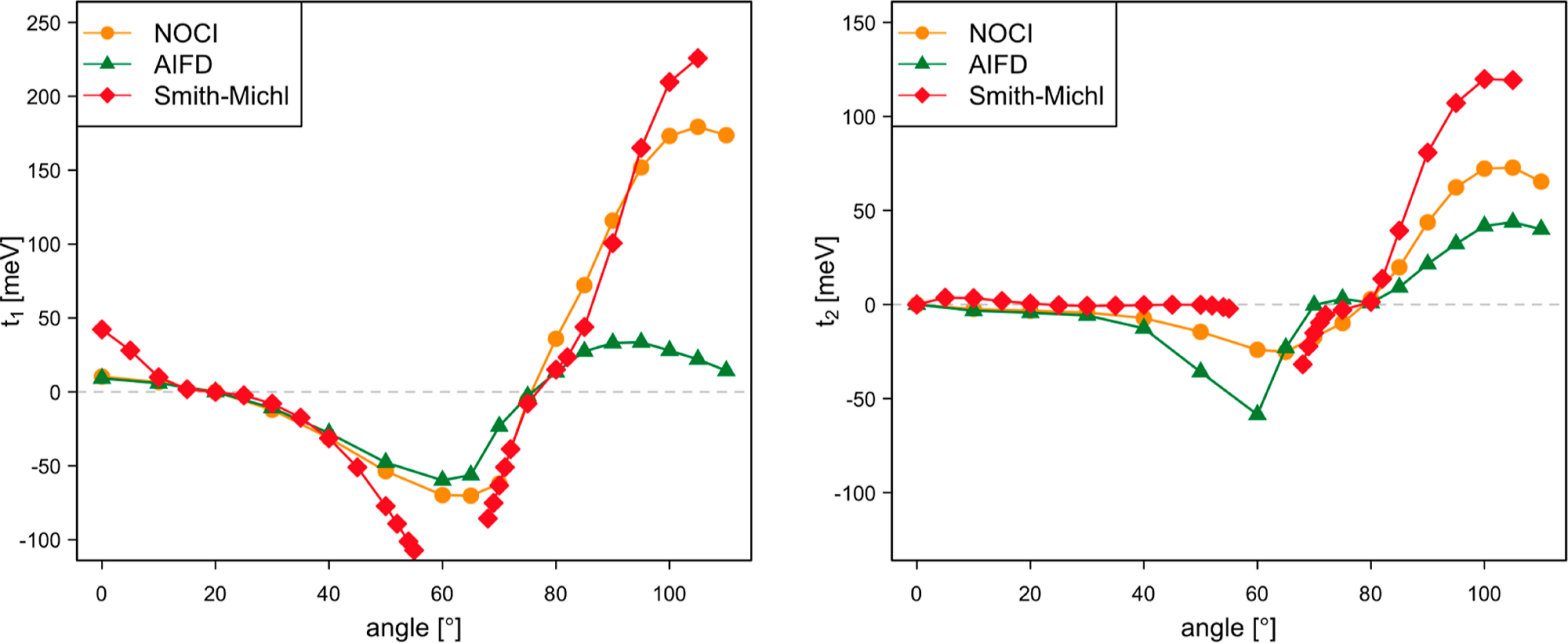
Singlet fission couplings *t*_1_ and *t*_2_ (in meV) as a function of the rotation angle of tetracene B.

By including the effect of the CT states (see Figure 15), the NOCI-F coupling strengths increase significantly, with *t*_1_ reaching almost 200 meV. The tendency observed for the NOCI-F direct coupling is more or less maintained, although the first extreme is shifted to larger rotation angles. The most striking difference with the direct coupling is the fact that the Smith–Michl model predicts couplings that are of the same order of magnitude and actually follow quite closely the NOCI-F results for most angles. Only in the interval between 55 and 68°, the model shows erratic behavior (see Figure S12), which can be ascribed to the fact that the CT MEBFs are nearly degenerate with the local excited singlet MEBFs in this region. The Smith–Michl model tends to overestimate the coupling, which is the most obvious for the largest angles considered here.

**Figure 16:**
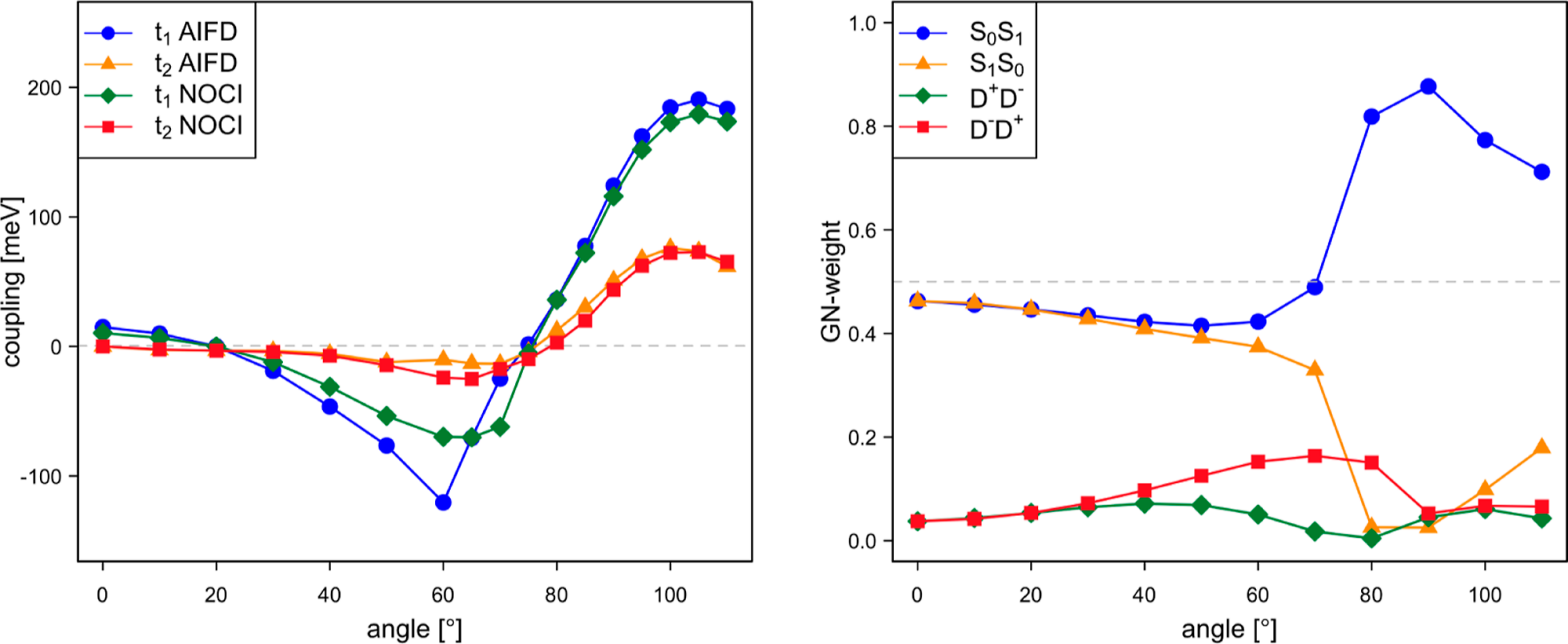
NOCI-F and shifted AIFD singlet fission couplings *t*_1_ and *t*_2_ (in meV) as a function of the rotation angle of tetracene B (left) and Gallup–Norbeck weights of the MEBFs in the AIFD initial state used to calculate *t*_1_ (right).

Although the incorporation of the CT effect increases the AIFD couplings, the final results are only in reasonable agreement with the NOCI-F values for angles smaller than 50°. Due to the approximate nature of the CIS and Δ*S*CF calculations applied in the AIFD scheme, the relative energies of the MEBFs deviate substantially from those in the NOCI-F calculations. In the present case, this is not only limited to the CT MEBFs as in dpp but also the ^1^TT MEBF shows a different relative energy. At zero angle, the ^1^TT lies 0.4 eV below the excited singlet exciton, while they are virtually degenerated in NOCI-F. The CT MEBFs lie about 0.18 eV lower in energy compared to NOCI-F. This leads to important changes in the calculated couplings as can be seen in the left panel of Figure 16, where the NOCI-F couplings are compared to those obtained with AIFD after shifting the energy of the MEBFs by 0.18 eV for D^+^D^–^ and D^–^D^+^ and 0.4 eV for ^1^TT. The sudden change in *t*_1_ around 70° reflects the change in the character of the MEBFs. The right panel displays the Gallup–Norbeck weights^45^ of the MEBFs in the singlet-dominated initial state used to calculate the *t*_1_ coupling in the left panel. For small angles, the singlet exciton is delocalized over both molecules, but between 60 and 80° rotation, the exciton localizes almost entirely on one of the fragments, accompanied by a more or less abrupt change in the dependence of *t*_1_ with the rotation angle.

## 6 Results: 5,5′-Difluoroindigo

The next system that is briefly discussed does not explore model geometries as the previous ones but focuses on an existing system of real interest, namely, the 5,5′-difluoroindigo compound. Its electronic features, notably, the measured transport properties, captured our attention, driving us to include this compound in the present study. The presence of two fluoro substituents confers on this molecule characteristic optical and electrochemical behavior which, of course, can be exploited in solar light harvesting, among other applications. In addition, the indigo family is more environmentally friendly than other organic semiconductors and stable enough to have potential as advanced materials.

**Figure 17:**
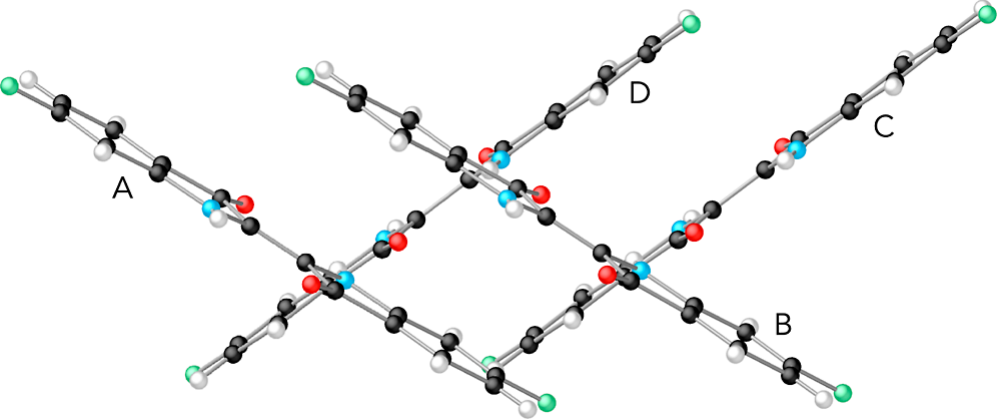
View of the 5,5′-difluoroindigo crystal structure along the *c*-axis of the unit cell.

This compound has been characterized as a crystal of the monoclinic system with *P*_21/*c*_ group,^34^ in which stacks of parallel units assemble, as represented in Figure 17. For a pair of neighboring parallel units (AB or CD in Figure 17), the relative positions are Δ*x* = 0.62 Å, Δ*y* = 5.01 Å, and Δ*z* = 3.34 Å, whereas a pair of oblique interstack units (AD or BC) form an interplanar angle of 67°. In this section, we explore the magnitude of different electronic pair properties for the two orientations, parallel or oblique.

The NOCI-F calculations are based on CASSCF fragment states with 10 active orbitals and 10 (S_0_, S_1_, and T_1_), 9 (D^+^) or 11 (D^–^) electrons. Figure S13 depicts the active orbitals of the S_1_ fragment state and is representative of all fragment states. The Δ*E* calculations are done with a small active space containing four orbitals and five electrons for electron transport and three electrons for hole transport. The rest of the methods follow the standard settings used before for dpp and tetracene. For DIPRO, we report only the values obtained with B3LYP KS orbitals.

The couplings for hole transport are similar in the two dimers, as shown in Table 2. The value in the parallel dimer is an order of magnitude smaller than that found for the perfectly stacked tetracene at Δ*z* = 3.3 Å, which shows that the sliding along *x* and certainly along *y* diminishes the coupling significantly. The angle between the molecular planes in the oblique dimer also prevents the coupling from becoming large. DIPRO and Δ*E* calculations follow the same tendency; they both predict smaller couplings as observed previously, although the underestimation of the DIPRO approach in the parallel dimer is more pronounced. The coupling involved in the electron transfer along the stacks (parallel dimers) does show up sizable, NOCI-F predicts a value of 120.8 meV, which could give rise to an efficient channel for electron transport. The interstack electron transfer does not seem to play an important role, a priori. Again DIPRO slightly underestimates the couplings, and Δ*E* gives rise to somewhat overestimated values.

**Table 2:** Electron Couplings (in meV) for 5,5′-Difluoroindigo Calculated with Different Computational Approaches

	NOCI-F	DIPRO	TDC	Δ*E*	Smith-Michl	AIFD
Parallel Dimer						
hole transport	9.5	0.7		2.9		
electron transport	120.8	66.7		176.5		
exciton transfer	20.0		22.2	29.4	25.0	78.2
singlet fission couplings	10.1				33.7	16.5
Oblique Dimer						
hole transport	14.3	13.1		9.6		
electron transport	8.7	7.9		18.4		
exciton transfer	19.5		4.1	23.5	31.2	12.5
singlet fission couplings	0.12				2.5	4.3

The exciton transfer is surprisingly constant along the different methods applied, exceptions are the overestimation by a factor of ∼4 for AIFD in the parallel dimer and the underestimation by TDC for the oblique one. Also, we observe that the total singlet fission coupling is significantly larger in the parallel dimer than that in the oblique one. In a perfectly stacked parallel dimer, this coupling would be strictly zero, but the sliding along the *x* and *y* directions lifts the symmetry and leads to nonzero coupling of the excited singlet with the singlet coupled double triplet. Both Smith–Michl and AIFD overestimate the coupling somewhat but do a proper job of predicting the relative strength of the coupling, in line with the observations for tetracene.

## 7 Benzene–Cl Complex

The excited state of the benzene–Cl atom complex represents a contact ion pair, benzene^+^–Cl^–^. Such contact ion pairs play an important role in the photochemical processes of organic molecules, which often take place at a femtosecond time scale.^46^ The electronic configuration of the ground state of the gas phase model system consists of a closed shell benzene plus a Cl-3p^5^ atom with an unpaired electron in the 3p_*z*_ orbital. Slightly higher in energy are the states with the holes in the 3p_*x*_ or 3p_*y*_ orbitals. The electron transfer from benzene to Cl creates a contact ion pair, consisting of two (nearly) degenerate electronic states, characterized by a closed shell Cl^–^ atom and a benzene cation with a hole in one of the highest occupied π orbitals. Cave and Newton studied the coupling for the electron transfer from benzene into the three Cl-3p orbitals as a function of the displacement of the Cl atom along the *x*-axis, parallel to the molecular benzene plane, see Figure 4.

**Figure 18:**
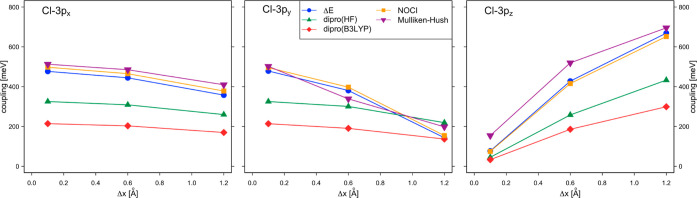
Electronic coupling (in meV) for electron transport in the benzene–Cl complex as a function of the displacement of the Cl atom along the *x*-axis.

SA-CASSCF(12,9) calculations were performed to calculate the dipole moments and relative energies of the five lowest doublet states at Δ*x* = 0.1, 0.6, and 1.2 Å. After the transition dipole moments between initial and final states were calculated, the Mulliken–Hush estimates of the coupling were determined (eq 9) and represented as purple inverted triangles in Figure 18. The active orbitals correspond to the six benzene π and three Cl-3p orbitals. While the coupling for the transfer into the Cl-3p_*x*_ orbital is relatively insensitive to the position of the Cl atom, the coupling for the other two transfer processes decreases (3*p*_*y*_) or increases (3*p*_*z*_) significantly. The values are similar to those found by Cave and Newton; the small differences are due to the differences in the computational settings.

The comparison with NOCI-F and the Δ*E*-based approaches can provide insight into what extent the different ways to construct the diabatic states affect the couplings. To stay as close as possible to the computational parameters used in the Mulliken–Hush approach, CASSCF(6,6) benzene wave functions are combined with restricted (open-shell) Hartree–Fock wave functions of Cl to generate the initial and final states of the different electron transfer processes. These NOCI-F estimates (orange squares) not only follow the same tendency as those obtained by the Mulliken–Hush procedure but are also quantitatively similar. The third approach for diabatization based on projection of the SA-CASSCF states on fragment localized wave functions (blue circles) is also close to the Mulliken–Hush results. The DIPRO approach (red diamonds and green triangles) predicts a similar tendency, but the couplings are in general smaller.

## 8 Conclusions

The results presented in the previous sections show that the approximate computational approaches can be used under certain circumstances as alternatives for NOCI-F to calculate electronic couplings in intermolecular energy and electron transport processes. The most straightforward case is provided by the electronic coupling for electron and hole transport. There are, in general, little differences between the outcomes of the methods applied. The NOCI-F couplings as a function of the intermolecular distance Δ*z* and the rotation angle are accurately reproduced by the DIPRO method and the Δ*E*-based calculations. This is probably caused by the fact that the couplings are neither sensitive to static electron correlation (no changes with increasing CAS) nor to dynamic electron correlation as switching off the correlation part of a hybrid functional does not affect the couplings.

Quantitively reproducing the NOCI-F electronic couplings for exciton transfer is more complicated, but TDC, Smith–Michl, and AIFD follow the trends predicted by NOCI-F. The couplings derived from the SA-CASSCF calculations on the dimer are more problematic. In some cases, the strong mixing of different electronic configurations leads to small projections on the model space and, hence, to unreliable results. TDC performs surprisingly well given its simplicity and the fact that the systems studied here are actually outside the normal application window of this approach, which is typically focused on chromophores that have stronger transition dipole moments and are further separated in space.

The most challenging case is posed by singlet fission coupling. The direct AIFD coupling compares well with that of NOCI-F, and the same holds for the Smith–Michl method for dpp, but it severely underestimates the coupling in tetracene. The situation is more complicated for the total coupling, that is, when the effect of the CT configurations is taken into account. AIFD suffers from the fact that the relative energies of the MEBFs are markedly different from those in the NOCI-F calculations. This leads to unexpected, irregular trends in the coupling, which are largely fixed by applying a constant shift on the diagonal matrix elements of the AIFD Hamiltonian. Aligning the relative energies of the MEBFs with the NOCI-F ones leads to total couplings that are in good agreement with those of NOCI-F over the whole range of angles. Smith–Michl does a reasonable job but has serious problems when the CT states are (nearly) degenerate with the S_0_S_1_ ± S_1_S_0_ or ^1^TT MEBFs. In these cases, the computed couplings are unreliable.

Since the electronic coupling between diabatic states of molecules or fragments is not observable, the computed coupling depends on the procedure used to define the initial and final states. The here compared approaches are based on three different schemes: (i) a combination of isolated fragment wave functions (NOCI-F, DIPRO, Smith–Michl, TDC, and AIFD), (ii) projection of adiabatic wave functions on fragments (the Δ*E* approach), and (iii) the dipole moment operator-based approach (Mulliken–Hush). Although there are certainly more ways of defining diabatic states and, moreover, the test systems only represent a small portion of all the possible situations, it can be stated as a first conclusion that the here-tested schemes do not lead to dramatically different outcomes. The largest differences are found in those cases in which the two fragments are very close to each other. Then, the Δ*E* approach appears to predict much larger couplings than the approaches based on combining fragment wave functions. It is difficult to say which estimate leads to better estimates of transition probabilities, but the fact is that these differences only show up in geometries that are physically not the most relevant ones. An intermolecular distance of 3.0 Å of two perfectly stacked organic molecules is quite unlikely given the high relative energy at this geometry; for example, it is more than 1 eV above the minimum around 4.5 Å for the dpp system.

## Supporting Information Available

Mathematical expressions of the Smith–Michl model, graphical representations of the active orbitals, electronic couplings as a function of the size of the active space, electronic couplings as a function of the density functional applied, electronic couplings as a function of the one electron basis set, decomposition of the DIPRO couplings for the hole and electron transport, decomposition of the TDC estimate for exciton coupling, Gallup–Norbeck weights of the MEBFs in the NOCI wave functions, and SA-CASSCF(8,8) energies of the five lowest excited singlet states of a tetracene dimer (PDF)

## References

[ref1] Straatsma T. P.; Broer R.; Sánchez-Mansilla A.; Sousa C.; de Graaf C. GronOR: Scalable and Accelerated Nonorthogonal Configuration Interaction for Molecular Fragment Wave Functions. J. Chem. Theory Comput. 2022, 18, 3549–3565. 10.1021/acs.jctc.2c00266. 35640094

[ref2] Sánchez-Mansilla A.; Sousa C.; Kathir R. K.; Broer R.; Straatsma T. P.; de Graaf C. On the role of dynamic electron correlation in non-orthogonal configuration interaction with fragments. Phys. Chem. Chem. Phys. 2022, 24, 11931–11944. 10.1039/D2CP00772J. 35521680

[ref3] Broer R.; Nieuwpoort W. C. Broken orbital symmetry and the description of valence hole states in the tetrahedral [CrO_4_]^2–^ anion. Theor. Chim. Acta 1988, 73, 405–418. 10.1007/BF00527744.

[ref4] Straatsma T. P.; Broer R.; Faraji S.; Havenith R. W. A.; Suarez L. E. A.; Kathir R. K.; Wibowo M.; de Graaf C. GronOR: Massively parallel and GPU-accelerated non-orthogonal configuration interaction for large molecular systems. J. Chem. Phys. 2020, 152, 064111 10.1063/1.5141358. 32061226

[ref5] Smith M. B.; Michl J. Singlet Fission. Chem. Rev. 2010, 110, 6891–6936. 10.1021/cr1002613. 21053979

[ref6] Casanova D. Theoretical modeling of singlet fission. Chem. Rev. 2018, 118, 7164–7207. 10.1021/acs.chemrev.7b00601. 29648797

[ref7] Morrison A. F.; You Z.-Q.; Herbert J. M. Ab Initio Implementation of the Frenkel-Davydov Exciton Model: A Naturally Parallelizable Approach to Computing Collective Excitations in Crystals and Aggregates. J. Chem. Theory Comput. 2014, 10, 5366–5376. 10.1021/ct500765m. 26583220

[ref8] Frenkel J. On the Transformation of light into Heat in Solids. I. Phys. Rev. 1931, 37, 17–44. 10.1103/PhysRev.37.17.

[ref9] Davydov A. S. Excitons in thin crystals. Phys.-Usp. 1964, 18, 496–499.

[ref10] Martin R. L. Natural transition orbitals. J. Chem. Phys. 2003, 118, 4775–4777. 10.1063/1.1558471.

[ref11] Mayer I. Using singular value decomposition for a compact presentation and improved interpretation of the CIS wave functions. Chem. Phys. Lett. 2007, 437, 284–286. 10.1016/j.cplett.2007.02.038.

[ref12] Plasser F.; Wormit M.; Dreuw A. New tools for the systematic analysis and visualization of electronic excitations. I. Formalism. J. Chem. Phys. 2014, 141, 024106 10.1063/1.4885819. 25027998

[ref13] Amos A. T.; Hall G. G. Single determinant wave functions. Proc. R. Soc. London, Ser. A 1961, 263, 483 10.1098/rspa.1961.0175.

[ref14] Smith M. B.; Michl J. Recent advances in singlet fission. Annu. Rev. Phys. Chem. 2013, 64, 361–386. 10.1146/annurev-physchem-040412-110130. 23298243

[ref15] Buchanan E. A.; Havlas Z.; Michl J. Singlet Fission: Optimization of Chromophore Dimer Geometry. Adv. Quantum Chem. 2017, 75, 175–227. 10.1016/bs.aiq.2017.03.005.

[ref16] Zaykov A.; Felkel P.; Buchanan E. A.; Jovanovic M.; Havenith R. W. A.; Kathir R. K.; Broer R.; Havlas Z.; Michl J. Singlet Fission Rate: Optimized Packing of a Molecular Pair. Ethylene as a Model. J. Am. Chem. Soc. 2019, 141, 17729–17743. 10.1021/jacs.9b08173. 31509712

[ref17] Löwdin P. O. On the non-orthogonality problem connected with the use of atomic wave functions in the theory of molecules and crystals. J. Chem. Phys. 1950, 18, 365–375. 10.1063/1.1747632.

[ref18] Baumeier B.; Kirkpatrick J.; Andrienko D. Density-functional based determination of intermolecular charge transfer properties for large-scale morphologies. Phys. Chem. Chem. Phys. 2010, 12, 11103–11113. 10.1039/c002337j. 20689881

[ref19] Bordas E.; Caballol R.; Graaf C. d.; Malrieu J.-P. Toward a variational treatment of the magnetic coupling between centers with elevated spin moments. Chem. Phys. 2005, 309, 259–269. 10.1016/j.chemphys.2004.09.016.

[ref20] de Graaf C.; Broer R. Magnetic Interactions in Molecules and Solids; Springer: Heidelberg, 2015.

[ref21] Krimm S.; Abe Y. Intermolecular Interaction Effects in the Amide I Vibrations of β Polypeptides. Proc. Natl. Acad. Sci. U.S.A. 1972, 69, 2788–2792. 10.1073/pnas.69.10.2788. 4507602 PMC389645

[ref22] Moore W. H.; Krimm S. Transition dipole coupling in AmideI modes of β polypeptides. Proc. Natl. Acad. Sci. U.S.A. 1975, 72, 4933–4935. 10.1073/pnas.72.12.4933. 16592297 PMC388847

[ref23] Torii H.; Tasumi M. Ab Initio Molecular Orbital Study of the Amide I Vibrational Interactions Between the Peptide Groups in Di- and Tripeptides and Considerations on the Conformation of the Extended Helix. J. Raman Spectrosc. 1998, 29, 81–86. 10.1002/(SICI)1097-4555(199801)29:1<81::AID-JRS214>3.0.CO;2-H.

[ref24] Kubelka J.; Kim J.; Bour P.; Keiderling T. A. Contribution of transition dipole coupling to amide coupling in IR spectra of peptide secondary structures. Vib. Spectrosc. 2006, 42, 63–73. 10.1016/j.vibspec.2006.04.003.

[ref25] la Cour Jansen T.; Dijkstra A. G.; Watson T. M.; Hirst D.; Knoester J. Modeling the amide I bands of small peptides. J. Chem. Phys. 2006, 125, 044312 10.1063/1.2218516. 16942147

[ref26] Baronio C. M.; Barth A. The Amide I Spectrum of Proteins—Optimization of Transition Dipole Coupling Parameters Using Density Functional Theory Calculations. J. Phys. Chem. B 2020, 124, 1703–1714. 10.1021/acs.jpcb.9b11793. 32040320 PMC7307917

[ref27] Jansen T. L. C. Computational spectroscopy of complex systems. J. Chem. Phys. 2021, 155, 170901 10.1063/5.0064092. 34742221

[ref28] Förster T. 10th Spiers Memorial Lecture. Transfer mechanisms of electronic excitation. Discuss. Faraday Soc. 1959, 27, 7–17. 10.1039/DF9592700007.

[ref29] Van Voorhis T.; Kowalczyk T.; Kaduk B.; Wang L.; Cheng C.-L.; Wu Q. The Diabatic Picture of Electron Transfer, Reaction Barriers, and Molecular Dynamics. Annu. Rev. Phys. Chem. 2010, 61, 149–170. 10.1146/annurev.physchem.012809.103324. 20055670

[ref30] Karman T.; Besemer M.; van der Avoird A.; Groenenboom G. C. Diabatic states, nonadiabatic coupling, and the counterpoise procedure for weakly interacting open-shell molecules. J. Chem. Phys. 2018, 148, 094105 10.1063/1.5013091.

[ref31] Yarkony D. R.; Xie C.; Zhu X.; Wang Y.; Malbon C. L.; Guo H. Diabatic and adiabatic representations: Electronic structure caveats. Comput. Theor. Chem. 2019, 1152, 41–52. 10.1016/j.comptc.2019.01.020.

[ref32] Cave R. J.; Newton M. D. Generalization of the Mulliken-Hush treatment for the calculation of electron transfer matrix elements. Chem. Phys. Lett. 1996, 249, 15–19. 10.1016/0009-2614(95)01310-5.

[ref33] Hsu C.-P. The Electronic Couplings in Electron Transfer and Excitation Energy Transfer. Acc. Chem. Res. 2009, 42, 509–518. 10.1021/ar800153f. 19215069

[ref34] Klimovich I. V.; Leshanskaya L. I.; Troyanov S. I.; Anokhin D. V.; Novikov D. V.; Piryazev A. A.; Ivanov D. A.; Dremova N. N.; Troshin P. A. Design of indigo derivatives as environment- friendly organic semiconductors for sustainable organic electronics. J. Mater. Chem. C 2014, 2, 7621–7631. 10.1039/C4TC00550C.

[ref35] Cervantes-Navarro F.; Glossman-Mitnik D. Density functional theory study of indigo and its derivatives as photosensitizers for dye-sensitized solar cells. J. Photochem. Photobiol., A 2013, 255, 24–26. 10.1016/j.jphotochem.2013.01.011.

[ref36] Li Manni G.; Fdez Galván I.; Alavi A.; Aleotti F.; Aquilante F.; Autschbach J.; Avagliano D.; Baiardi A.; Bao J. J.; Battaglia S.; et al. The OpenMolcas Web: A Community-Driven Approach to Advancing Computational Chemistry. J. Chem. Theory Comput. 2023, 19, 6933–6991. 10.1021/acs.jctc.3c00182. 37216210 PMC10601490

[ref37] Kathir R. K.; de Graaf C.; Broer R.; Havenith R. W. A. Reduced Common Molecular Orbital Basis for Nonorthogonal Configuration Interaction. J. Chem. Theory Comput. 2020, 16, 2941–2951. 10.1021/acs.jctc.9b01144. 32279493 PMC7222100

[ref38] Lehtola S.; Steigemann C.; Oliveira M. J. T.; Marques M. A. L. Recent developments in Libxc—A comprehensive library of functionals for density functional theory. Software X 2018, 7, 1–5. 10.1016/j.softx.2017.11.002.

[ref39] Aquilante F.; Lindh R.; Bondo Pedersen T. Unbiased auxiliary basis sets for accurate two-electron integral approximations. J. Chem. Phys. 2007, 127, 114107 10.1063/1.2777146. 17887828

[ref40] de Graaf C.; Illas F. Electronic structure and magnetic interactions of the spin chain compounds Ca_2_CuO_3_ and Sr_2_CuO_3_. Phys. Rev. B: Condens. Matter Mater. Phys. 2000, 63, 014404 10.1103/physrevb.63.014404.

[ref41] Bryenton K. R.; Adeleke A. A.; Dale S. G.; Johnson E. R. Delocalization error: The greatest outstanding challenge in density-functional theory. Wiley Interdiscip. Rev.: Comput. Mol. Sci. 2023, 13, e1631 10.1002/wcms.1631.

[ref42] Bao J. L.; Gagliardi L.; Truhlar D. G. Self-Interaction Error in Density Functional Theory: An Appraisal. J. Phys. Chem. Lett. 2018, 9, 2353–2358. 10.1021/acs.jpclett.8b00242. 29624392

[ref43] Pandharkar R.; Hermes M. R.; Cramer C. J.; Gagliardi L. Localized Active Space-State Interaction: a Multireference Method for Chemical Insight. J. Chem. Theory Comput. 2022, 18, 6557–6566. 10.1021/acs.jctc.2c00536. 36257065

[ref44] Farag M. H.; Krylov A. I. Singlet Fission in Perylenediimide Dimers. J. Phys. Chem. C 2018, 122, 25753–25763. 10.1021/acs.jpcc.8b05309.

[ref45] Gallup G. A.; Norbeck J. M. Population analyses of valence-bond wavefunctions and BeH_2_. Chem. Phys. Lett. 1973, 21, 495–500. 10.1016/0009-2614(73)80292-1.

[ref46] Kumpulainen T.; Lang B.; Rosspeintner A.; Vauthey E. Ultrafast Elementary Photochemical Processes of Organic Molecules in Liquid Solution. Chem. Rev. 2017, 117, 10826–10939. 10.1021/acs.chemrev.6b00491. 27957848

