# Supplementary material for: Comparison of Computational Strategies for the Calculation of the Electronic Coupling in Intermolecular Energy and Electron Transport Processes
Source: J Phys Chem A. 2023 Dec 12;127(50):10717–31. doi: 10.1021/acs.jpca.3c05998 (PMC10749449; doi:10.1021/acs.jpca.3c05998)
Supplement: Supplementary file 1 — jp3c05998_si_001.pdf [file jp3c05998_si_001.pdf]

Supporting Information for "On the Comparison of Computational Strategies for the Calculation of the Electronic Coupling in Intermolecular Energy and Electron Transport Processes"

Xavier López,[†] Aitor Sánchez-Mansilla,[†] Carmen Sousa,[‡] Tjerk P. Straatsma,[¶,§] Ria Broer,[∥] and Coen de Graaf[*,⊥,#]

†*Departament de Química Física i Inorgànica, Universitat Rovira i Virgili, Spain*

‡*Departament de Ciència de Materials i Química Física and Institut de Química Teòrica i Computacional, Universitat de Barcelona, Spain*

¶*National Center for Computational Sciences, Oak Ridge National Laboratory, Oak Ridge, TN 37831-6373, U. S. A.*

§*Department of Chemistry and Biochemistry, University of Alabama, Tuscaloosa, AL 35487-0336, U. S. A.*

∥*Zernike Institute of Advanced Materials, University of Groningen, Netherlands*

⊥*Departament de Química Física i Inorgànica, Universitat Rovira i Virgili.*

#*ICREA, Pg. Lluís Companys 23, Barcelona, Spain*

E-mail: coen.degraaf@urv.cat

1 Energy expressions and interactions for the Smith-Michl model

In the following, a represents the HOMO on molecule A, b the HOMO on B, c and d are the LUMO orbitals on molecule A and B, respectively. Furthermore, h_{ij} stands for $\langle i|\hat{h}|j\rangle$ with $\hat{h}$ the one-electron operator and $\langle ij||kl\rangle$ is the short-hand notation for $\langle ij|\frac{1}{r_{12}}|kl\rangle$. The overlined orbitals have electrons with β spin, the others represent electrons with α spin.

1. Definition of the electronic states:

$$
\begin{aligned}
|S_0S_0\rangle &= |a\bar{a}b\bar{b}| \\
|S_0S_1\rangle &= \frac{1}{\sqrt{2}}(|a\bar{a}b\bar{d}| + |a\bar{a}d\bar{b}|) \\
|S_1S_0\rangle &= \frac{1}{\sqrt{2}}(|a\bar{c}b\bar{b}| + |c\bar{a}b\bar{b}|) \\
|T_1T_1\rangle &= \frac{1}{\sqrt{3}}\{|a\bar{b}c\bar{d}| + |b\bar{a}d\bar{c}| + \frac{1}{2}(|a\bar{c}b\bar{d}| - |a\bar{c}d\bar{b}| - |c\bar{a}b\bar{d}| + |c\bar{a}d\bar{b}|)\} \\
\left|D^+D^-\right\rangle &= \frac{1}{\sqrt{2}}(|a\bar{d}b\bar{b}| + |d\bar{a}b\bar{b}|) \\
\left|D^+D^-\right\rangle &= \frac{1}{\sqrt{2}}(|a\bar{a}b\bar{c}| + |a\bar{a}c\bar{b}|)
\end{aligned}
$$

2. Diagonal matrix elements of the Hamiltonian

$$\langle S_0S_0|\hat{H}|S_0S_0\rangle = 2h_{aa} + 2h_{bb} + \langle aa||aa\rangle + \langle bb||bb\rangle + 4\langle ab||ab\rangle - 2\langle ab||ba\rangle$$

$$\langle S_0S_1|\hat{H}|S_0S_1\rangle = 2h_{aa} + h_{bb} + h_{dd} + \langle aa||aa\rangle + 2\langle ab||ab\rangle + 2\langle ad||ad\rangle + \langle bd||bd\rangle$$
$$- \langle ad||da\rangle - \langle ab||ba\rangle + \langle bd||db\rangle$$

$$\langle S_1S_0|\hat{H}|S_1S_0\rangle = h_{aa} + 2h_{bb} + h_{cc} + \langle bb||bb\rangle + 2\langle ab||ab\rangle + 2\langle bc||bc\rangle + \langle ac||ac\rangle$$
$$- \langle bc||cb\rangle - \langle ab||ba\rangle + \langle ac||ca\rangle$$

$$\langle T_1T_1|\hat{H}|T_1T_1\rangle = h_{aa} + h_{bb} + h_{cc} + h_{dd} + \langle ab||ab\rangle + \langle ac||ac\rangle + 2\langle ad||ad\rangle$$
$$+ \langle bc||bc\rangle + \langle bd||bd\rangle + \langle cd||cd\rangle - \langle ac||ca\rangle - \langle bd||db\rangle$$

$$\left\langle D^+D^-\middle|\hat{H}\middle|D^+D^-\right\rangle = h_{aa} + 2h_{bb} + h_{dd} + 2\langle ab||ab\rangle + \langle ad||ad\rangle + \langle bb||bb\rangle$$
$$+2\langle bd||bd\rangle - \langle ab||ba\rangle - \langle bd||db\rangle$$

$$\left\langle D^-D^+\middle|\hat{H}\middle|D^-D^+\right\rangle = 2h_{aa} + h_{bb} + h_{cc} + \langle aa||aa\rangle + \langle ac||ac\rangle + 2\langle ab||ab\rangle$$
$$+2\langle ac||ac\rangle + \langle bc||bc\rangle - \langle ab||ba\rangle - \langle ac||ca\rangle$$

3. Off-diagonal elements of the Hamiltonian

$$\langle S_0S_1|\hat{H}|S_1S_0\rangle = 2\langle ad||cb\rangle - \langle ad||bc\rangle$$

$$\langle S_0S_1|\hat{H}|T_1T_1\rangle = \sqrt{\tfrac{3}{2}}\big(\langle ab||bc\rangle - \langle ad||dc\rangle\big)$$

$$\big\langle S_0S_1\big|\hat{H}\big|D^+D^-\big\rangle = -h_{ab} - \langle aa||ab\rangle - \langle ab||bb\rangle - \langle ad||bd\rangle + 2\langle ad||db\rangle$$

$$\big\langle S_0S_1\big|\hat{H}\big|D^-D^+\big\rangle = h_{cd} + 2\langle ad||ac\rangle - \langle ad||ca\rangle + \langle bd||bc\rangle + \langle bd||cb\rangle$$

$$\langle S_1S_0|\hat{H}|T_1T_1\rangle = \sqrt{\tfrac{3}{2}}\big(\langle ab||da\rangle - \langle cb||dc\rangle\big)$$

$$\big\langle S_1S_0\big|\hat{H}\big|D^+D^-\big\rangle = h_{cd} + \langle ac||ad\rangle + 2\langle cb||db\rangle - \langle cb||bd\rangle + \langle ac||da\rangle$$

$$\big\langle S_1S_0\big|\hat{H}\big|D^-D^+\big\rangle = -h_{ab} + 2\langle cb||ac\rangle - \langle ab||aa\rangle - \langle cb||ca\rangle - \langle bb||ab\rangle$$

$$\big\langle T_1T_1\big|\hat{H}\big|D^+D^-\big\rangle = \sqrt{\tfrac{3}{2}}\big(h_{bc} + \langle ab||ac\rangle - \langle ab||ca\rangle + \langle db||dc\rangle + \langle bb||bc\rangle\big)$$

$$\big\langle T_1T_1\big|\hat{H}\big|D^-D^+\big\rangle = \sqrt{\tfrac{3}{2}}\big(h_{ad} + \langle aa||ad\rangle + \langle ac||dc\rangle + \langle ab||db\rangle - \langle ab||bd\rangle\big)$$

$$\big\langle D^+D^-\big|\hat{H}\big|D^-D^+\big\rangle = 2\langle db||ac\rangle - \langle db||ca\rangle$$

2 Results: Diketopyrrolopyrol (dpp)

2.1 NOCI couplings for electron and hole transport

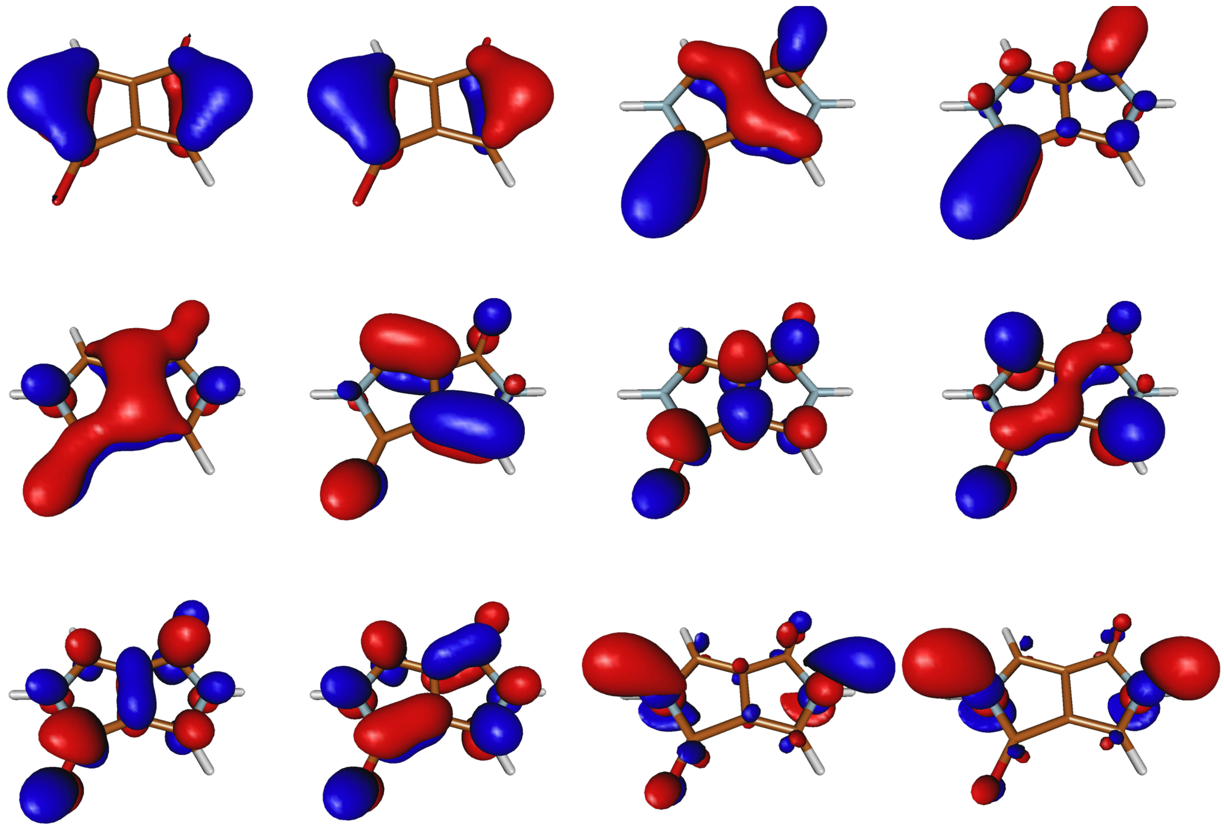

Figure S1: Active orbitals of the CAS(12,12) calculation of the S_0 electronic state of dpp. The orbitals of the smaller active space are similar and are obtained by removing one by one the first and the last active orbital from the previous (larger) active space.

Table S1: NOCI electronic couplings for hole transport as function of the intermolecular distance (in Å) of two perfectly stacked parallel dpp molecules for different active spaces

Δz	CAS(12,12)	CAS(10,10)	CAS(8,8)	CAS(6,6)	CAS(4,4)
3.0	850	809	803	759	831
3.5	442	423	400	373	412
4.0	228	220	198	183	202
4.5	118	114	98	89	99
5.0	62	60	49	44	49
5.5	33	32	25	22	25
6.0	17	17	13	11	13

Table S2: NOCI electronic couplings for electron transport as function of the intermolecular distance (in Å) of two perfectly stacked parallel dpp molecules for different active spaces

Δz	CAS(12,12)	CAS(10,10)	CAS(8,8)	CAS(6,6)	CAS(4,4)
3.0	749	772	773	796	808
3.5	393	412	410	422	430
4.0	209	224	221	227	232
4.5	113	123	121	123	127
5.0	62	69	68	69	71
5.5	35	39	39	39	41
6.0	20	23	22	22	23

2.2 DIPRO couplings for electron and hole transport

Table S3: DIPRO electron hopping parameter (in meV) for two dpp molecules as function of the intermolecular distance along the z-axis for different functionals.

Δz [Å]	HF	SSBD	B3LYP	M062X	PBE	LDA	M06HF	HFB86
3.0	875	573	644	737	585	602	831	573
3.5	475	267	312	374	273	282	445	280
4.0	268	127	153	185	131	134	245	142
4.5	156	61	76	93	63	63	136	74
5.0	93	30	38	49	31	30	76	40
5.5	57	15	19	27	16	14	44	22
6.0	35	7	10	15	8	7	26	12

Table S4: DIPRO hole hopping parameter (in meV) for two dpp molecules as function of the intermolecular distance along the z-axis for different functionals.

Δz [Å]	HF	SSBD	B3LYP	M062X	PBE	LDA	M06HF	HFB86
3.0	827	512	577	625	524	540	753	515
3.5	397	226	264	308	233	242	358	237
4.0	190	101	121	142	104	108	170	111
4.5	89	45	54	62	47	48	80	53
5.0	41	20	24	27	21	21	38	25
5.5	19	9	11	13	9	9	17	12
6.0	9	4	5	6	4	4	8	6

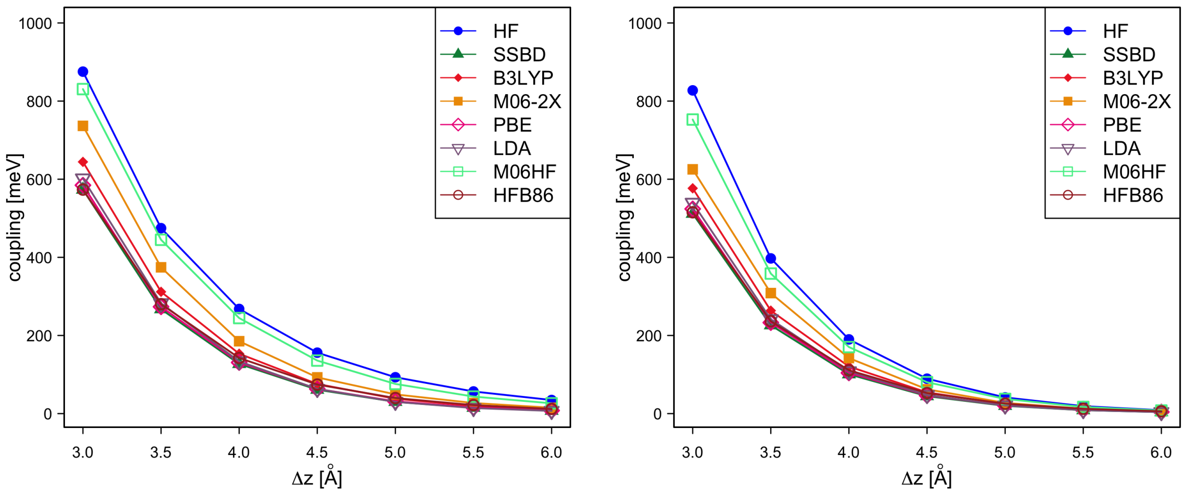

Figure S2: DIPRO electron (left) and hole (right) hopping parameter for two dpp molecules as function of the intermolecular distance along the z-axis for different functionals.

Table S5: Decomposition of the DIPRO coupling for electron transport between two perfectly stacked parallel dpp molecules (Δz = 3.0 Å) as function of the amount of Fock exchange (α) in the hybrid functional $\alpha[\rho_x^{HF}]+(1-\alpha)[\rho_x^{B88}]+\beta[\rho_C^{LYP}]$ ($\beta = 1$). J_{AB} and γ_{if}^{el} are given in meV, ϵ_A, ϵ_B in eV.

	hole				electron			
α	J_{AB}	S_{AB}	ϵ_A, ϵ_B	γ_{if}^{el}	J_{AB}	S_{AB}	ϵ_A, ϵ_B	γ_{if}^{el}
0.9	1670	-0.1006	-8.77	796	930	-0.1362	-0.47	883
0.8	1581	-0.0987	-8.36	763	939	-0.1316	-0.80	849
0.7	1494	-0.0969	-7.95	731	944	-0.1269	-1.12	815
0.6	1408	-0.0949	-7.54	699	945	-0.1222	-1.44	781
0.5	1325	-0.0929	-7.14	667	943	-0.1175	-1.76	746
0.4	1244	-0.0909	-6.75	637	936	-0.1129	-2.06	712
0.3	1166	-0.0887	-6.36	606	926	-0.1083	-2.36	678
0.2	1090	-0.0866	-5.97	577	913	-0.1038	-2.65	645
0.1	1017	-0.0844	-5.59	549	897	-0.0993	-2.94	612

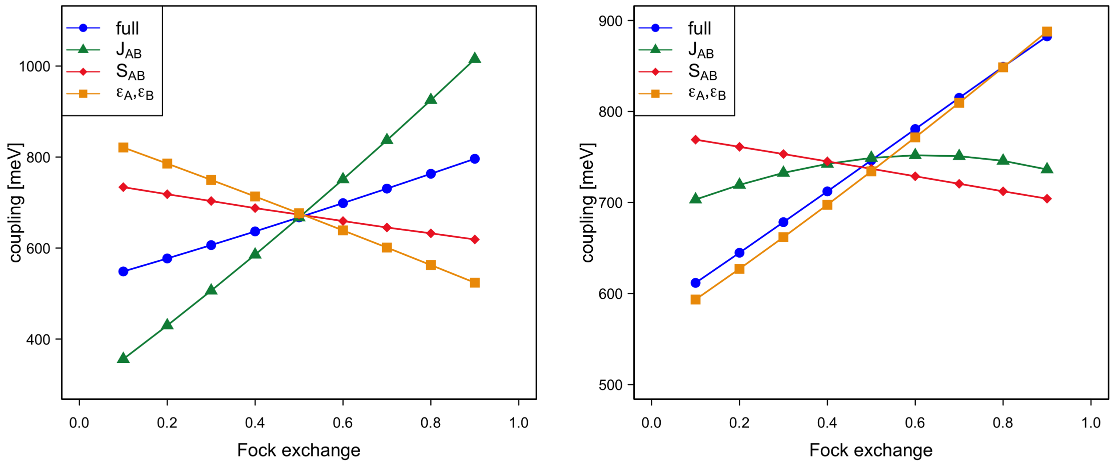

Figure S3: Effect of J_{AB}, S_{AB} and $\epsilon_{A,B}$ on the DIPRO coupling for hole (left) and electron (right) transport between two perfectly stacked parallel dpp molecules (Δz = 3.0 Å) as function of the amount of Fock exchange (α) in the hybrid functional $\alpha[\rho_x^{HF}]+(1-\alpha)[\rho_x^{B88}]+\beta[\rho_C^{LYP}]$ ($\beta = 1$). The blue curve represents the γ_{if}^{el}-values listed in Table S5. The green curve uses the J_{AB}-values of Table S5, but fixes S_{AB} and the orbital energies to their average values. The other two curves were obtained with varying S_{AB} (red) or varying orbital energies (orange), fixing the other two variables to their average values.

2.3 Exciton coupling

Table S6: NOCI electronic couplings (in meV) for exciton transport as function of the intermolecular distance (in Å) of two perfectly stacked parallel dpp molecules for different active spaces

Δz	CAS(12,12)	CAS(10,10)	CAS(8,8)	CAS(6,6)	CAS(4,4)
3.0	336	336	374	374	429
3.5	198	196	215	205	248
4.0	133	131	141	124	162
4.5	98	96	101	80	116
5.0	75	73	76	52	87
5.5	59	58	60	32	67
6.0	48	46	47	18	53

Table S7: ΔE-based electronic couplings (in meV) for exciton transport as function of the intermolecular distance (in Å) of two perfectly stacked parallel dpp molecules for different active spaces

Δz	CAS(4,4)	CAS(8,8)	CAS(12,12)	CAS(16,16)
3.0	982	955	826	761
3.5	524	510	380	434
4.0	279	262	190	217
4.5	169	154	118	120
5.0	119	108	86	92
5.5	90	82	66	70
6.0	71	64	53	53

Table S8: TDC for exciton transport (in meV) as function of the intermolecular distance (in Å) of two perfectly stacked parallel dpp molecules for different active spaces

Δz	CAS(2,2)	CAS(4,4)	CAS(6,6)	CAS(8,8)	CAS(10,10)	CAS(12,12)
3.0	785	558	608	510	499	516
3.5	494	351	383	321	314	325
4.0	331	235	257	215	211	218
4.5	233	165	180	151	148	153
5.0	170	120	131	110	108	111
5.5	127	91	99	83	81	84
6.0	98	70	76	64	62	64

Table S9: Transition dipole moment of the $S_0 \rightarrow S_1$ excitation of a dpp molecule for different active spaces

	μ_x	μ_y	μ_z	Total
CAS(2,2)	-0.05	2.29	0.00	5.26
CAS(4,4)	-0.18	1.92	0.00	3.73
CAS(6,6)	-0.45	1.97	0.00	4.07
CAS(8,8)	0.25	-1.83	0.00	3.42
CAS(10,10)	-0.49	1.76	0.00	3.34
CAS(12,12)	-0.63	1.75	0.00	3.45

Table S10: TDC for exciton transport (in meV) as function of the intermolecular distance (in Å), with dpp molecule B displaced by $\mathbf{\Delta x = \Delta y = 1.0}$Å

Δz	$\frac{\vec{\mu}_A \cdot \vec{\mu}_B}{r_{AB}^3}$	$-3\frac{\vec{\mu}_A \cdot \vec{r}_{AB} \vec{\mu}_B \cdot \vec{r}_{AB}}{r_{AB}^5}$	TDC
3.0	377.5	-74.8	302.7
3.5	256.0	-39.2	216.9
4.0	180.3	-21.8	158.5
4.5	131.2	-12.9	118.4
5.0	98.2	-7.9	90.2
5.5	75.2	-5.1	70.1
6.0	58.8	-3.4	55.4

Table S11: TDC for exciton transport (in meV) as function of the rotation angle of dpp molecule B, displaced by $\mathbf{\Delta x = \Delta y = 1.0Å}$, $\mathbf{\Delta z = 4.5Å}$.

angle	$\frac{\vec{\mu}_A \cdot \vec{\mu}_B}{r_{AB}^3}$	$-3\frac{\vec{\mu}_A \cdot \vec{r}_{AB} \vec{\mu}_B \cdot \vec{r}_{AB}}{r_{AB}^5}$	TDC
0	131.2	-12.9	118.4
10	129.2	-9.7	119.5
20	123.3	-6.3	117.1
30	113.6	-2.6	111.0
40	100.5	1.1	101.6
50	84.4	4.8	89.1
60	65.6	8.3	73.9
70	44.9	11.6	56.5
80	22.8	14.5	37.3
90	0.0	17.0	17.0

Table S12: Transition dipole moment of the $S_0 \rightarrow S_1$ excitation of a dpp molecule for three different ANO-RCC one-electron basis sets using a CAS(12,12)

C, N, O	H	μ_x	μ_y	μ_z	Total
3s, 2p, 1d	2s, 1p	0.69	-1.72	0.00	3.44
4s, 3p, 2d, 1f	3s, 2p, 1d	0.62	1.71	0.00	3.32
5s, 4p, 3d, 2f	4s, 3p, 2d	0.60	1.71	0.00	3.29

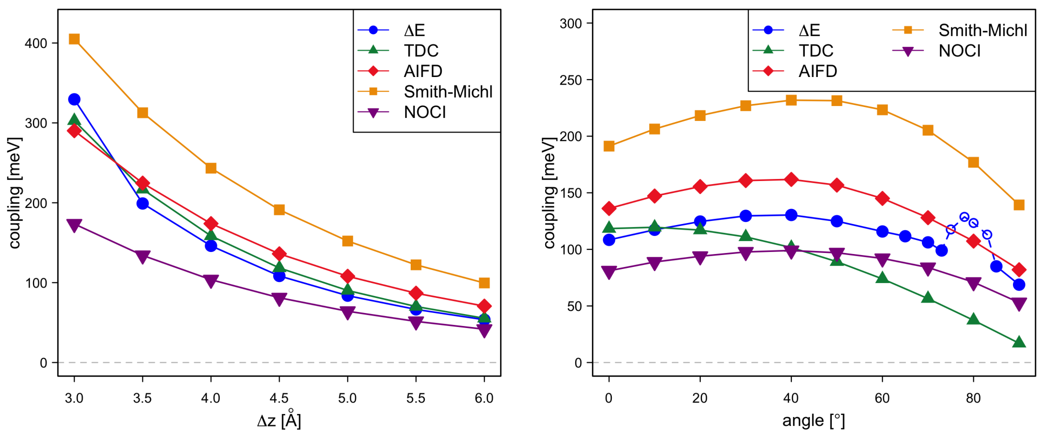

Figure S4: Electronic coupling (in meV) for exciton transport as function of the intermolecular distance of parallel dpp molecules (left) and as of function of the rotation angle of the second dpp molecule (right), which is displaced by $\Delta x = \Delta y = 1.0$ Å, $\Delta z = 4.5$ Å.

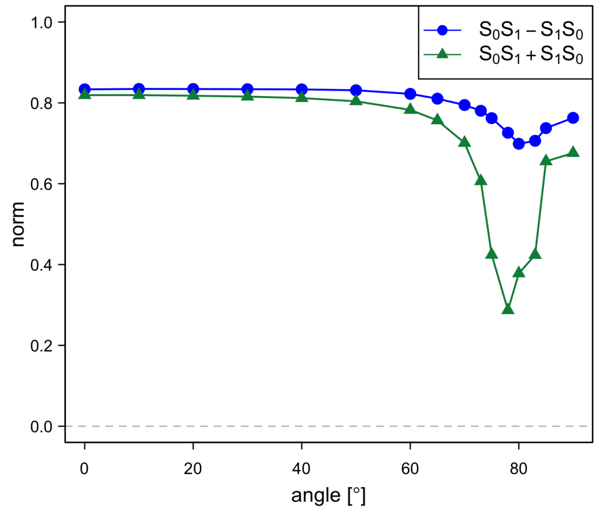

Figure S5: Norm of the largest $S_0S_1 \pm S_1S_0$ projections on the lowest eight roots of the CASSCF(8,8) calculation of a dpp dimer as function of the rotation of dpp molecule B, displaced by $\Delta x = \Delta y = 1.0$ Å, $\Delta z = 4.5$ Å.

2.4 Singlet fission coupling

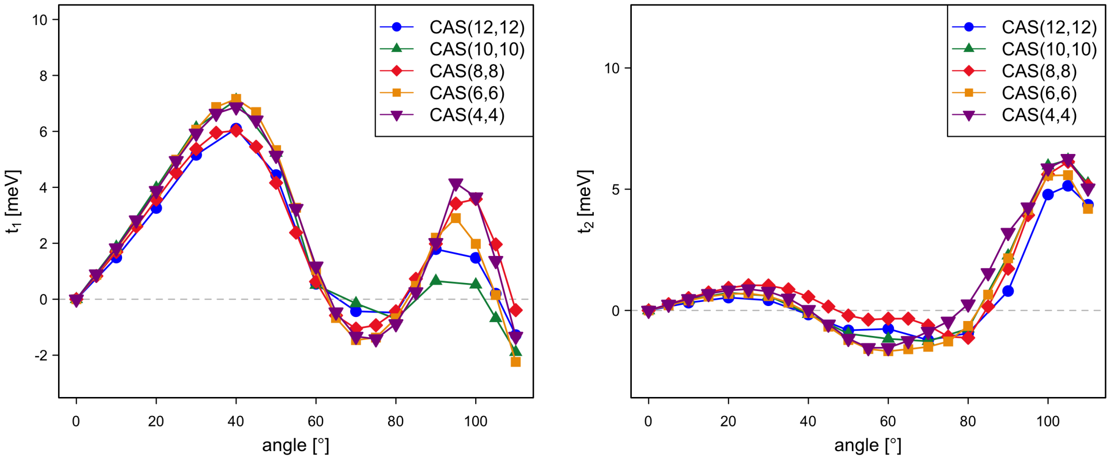

Figure S6: NOCI direct singlet fission couplings t_1 (left) and t_2 (right) (in meV) applying different complete active spaces for the fragment wave functions

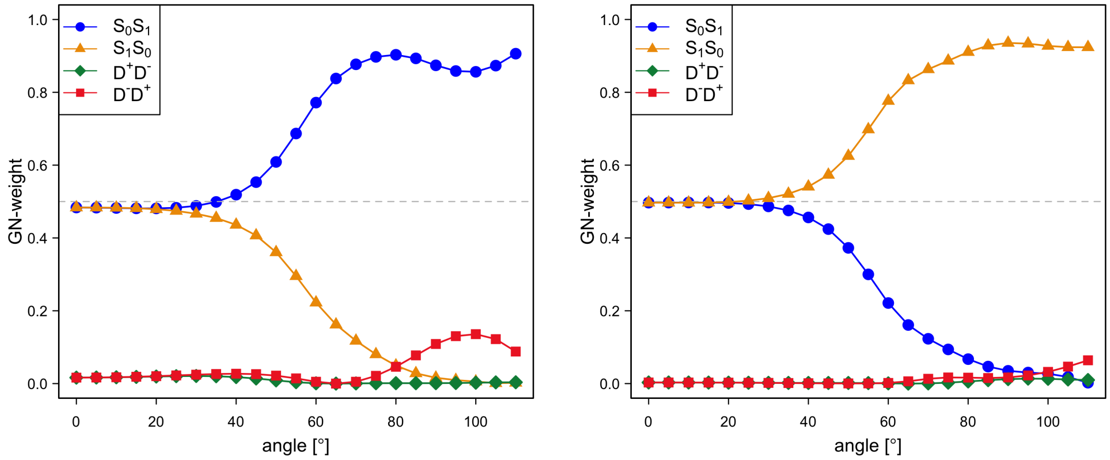

Figure S7: Gallup-Norbeck weights of the $S_0S_1 \pm S_1S_0$ dominated MEBFs used to calculate the NOCI total singlet fission couplings t_1 (left) and t_2 (right).

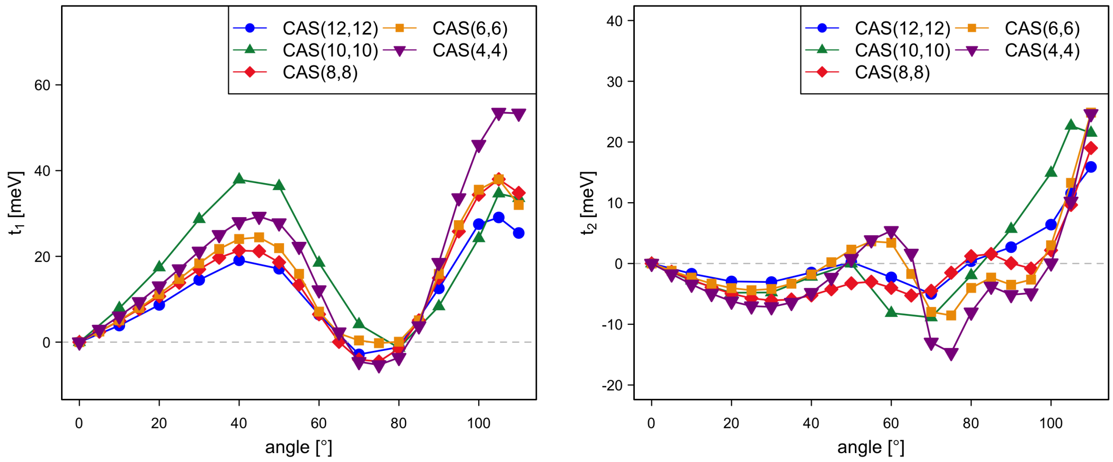

Figure S8: NOCI total singlet fission couplings t_1 (left) and t_2 (right) (in meV) applying different complete active spaces for the fragment wave functions

3 Results: Tetracene

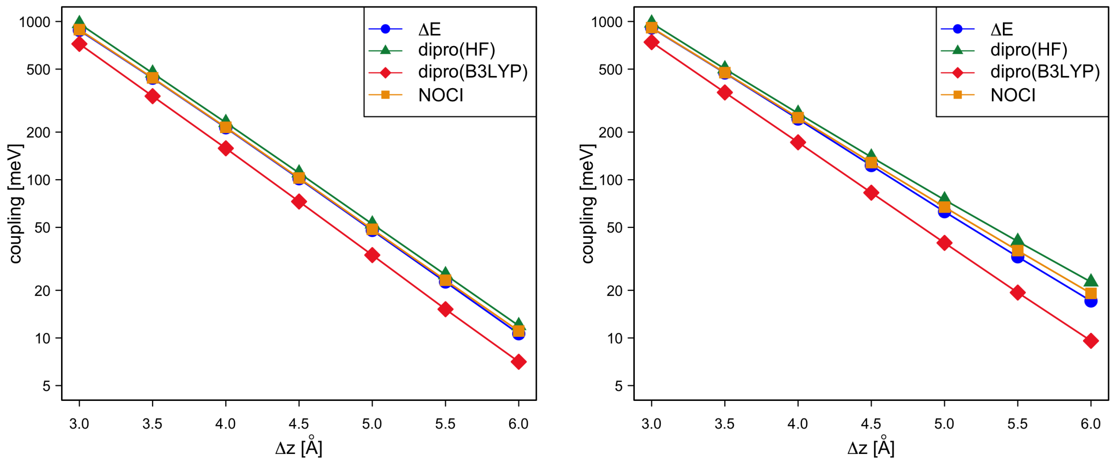

Figure S9: Electronic coupling (in meV) for hole transport (left) and electron transport (right) as function of the intermolecular distance of two perfectly stacked parallel tetracene molecules

S_1 CASSCF(10,10) active orbitals for tetracene molecule

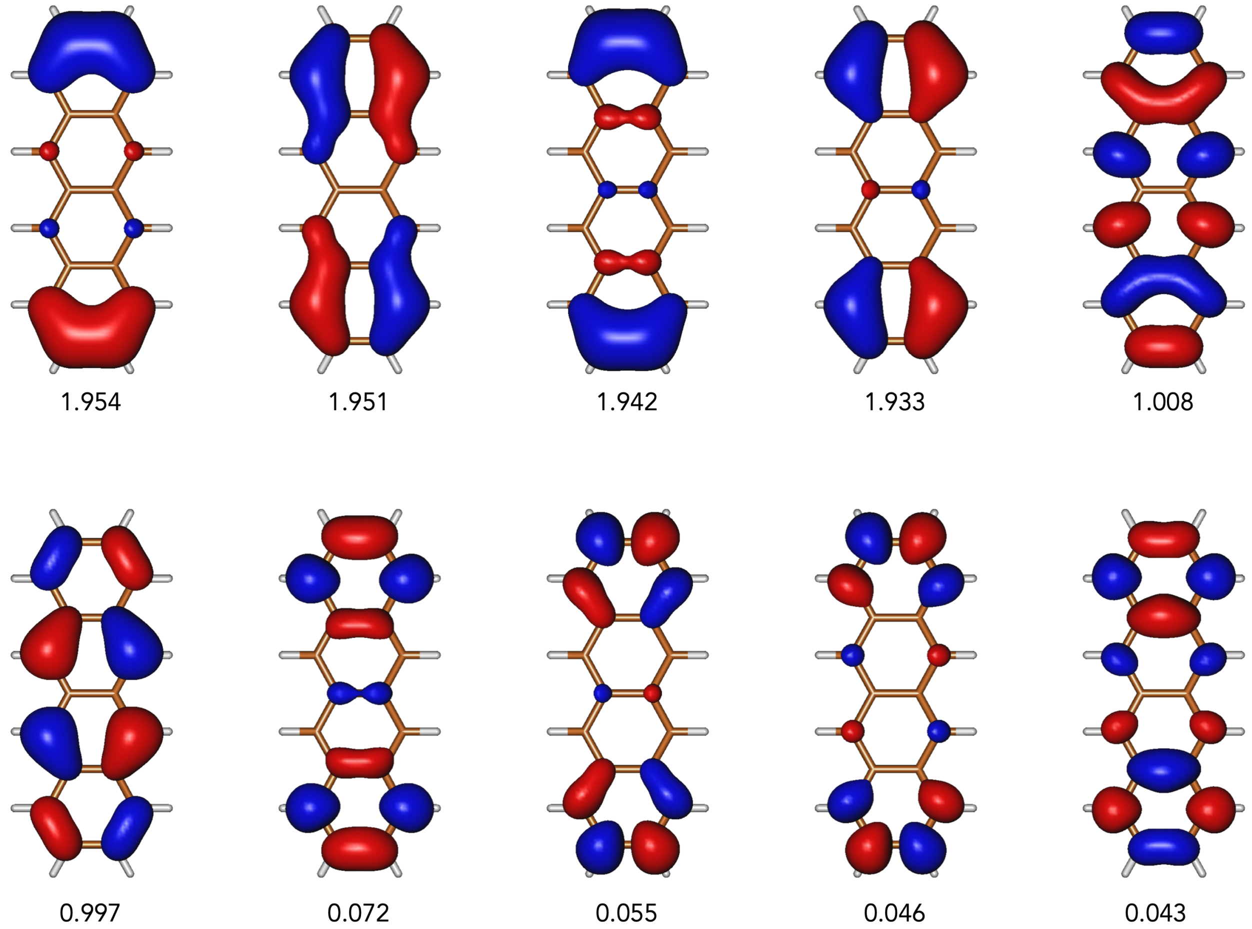

Figure S10: Active orbitals and natural occupation numbers of the CAS(10,10) calculation of the S_1 electronic state of tetracene. The orbitals of the other fragment states are similar in shape.

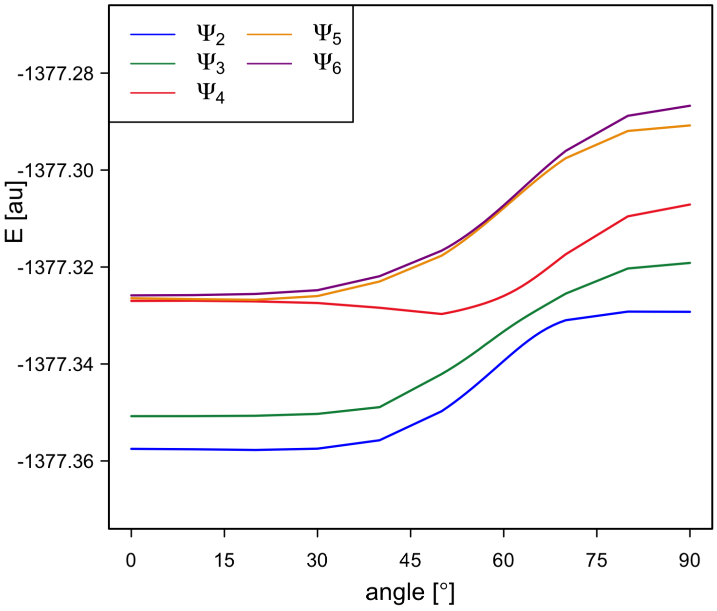

Figure S11: SA-CASSCF(8,8) energies of the five lowest excited singlet states of a tetracene dimer as function of the rotation angle of tetracene B.

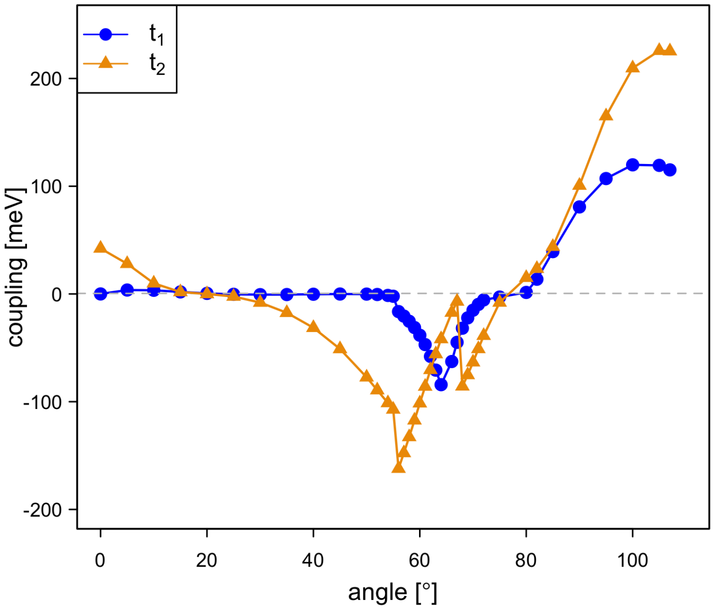

Figure S12: Smith-Michl total singlet fission couplings of a tetracene dimer as function of the rotation angle of tetracene B.

4 5,5'-difluoroindigo

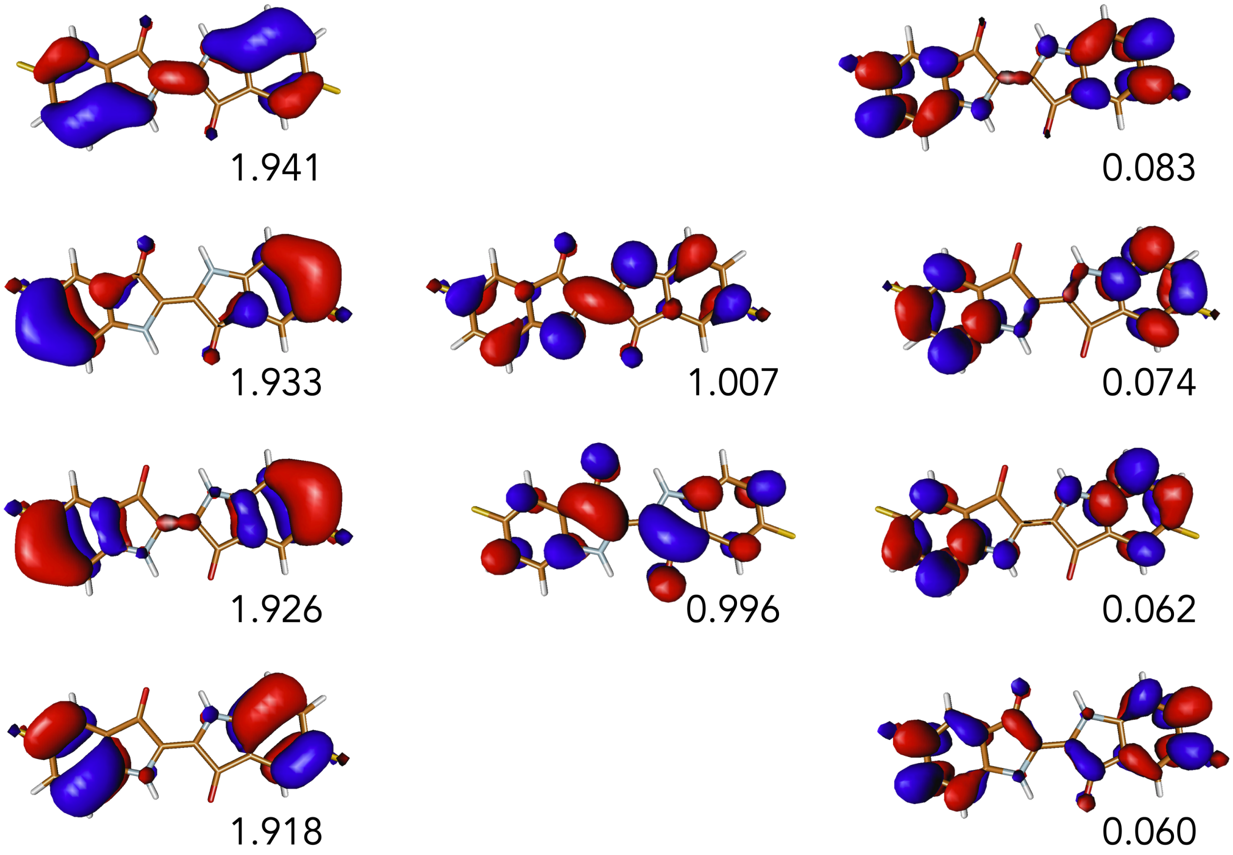

Figure S13: Active orbitals and natural occupation numbers of the CAS(10,10) calculation of the S_1 electronic state of 5,5'-difluoroindigo. The orbitals of the other fragment states are similar in shape.
